# Integrated transcriptomic, proteomic, and metabolomic analysis unveils key roles of protein and nucleic acid interactions in diabetic ulcer pathogenesis

**DOI:** 10.3389/fendo.2025.1574858

**Published:** 2025-06-20

**Authors:** Yongpan Lu, Hairui Gao, Sen Wang, Han Xu, Zhiyu Chen, Yixin Zhang, Yunfei Gu, Xiaomei Sun

**Affiliations:** ^1^ Department of Anorectal Surgery, The Affiliated Hospital of Qingdao University, Qingdao, Shandong, China; ^2^ Department of Plastic Surgery, The First Affiliated Hospital of Shandong First Medical University & Shandong Provincial Qianfoshan Hospital, Jinan, Shandong, China; ^3^ Department of Ultrasound, Beijing Tiantan Hospital, Capital Medical University, Beijing, China; ^4^ Department of Radiology, Qilu Hospital of Shandong University, Jinan, China; ^5^ Department of Ultrasound, Sun Yat-sen Memorial Hospital, Sun Yat-sen University, Guangzhou, Guangdong, China; ^6^ Department of Colorectal Surgery, The Affiliated Hospital of Nanjing University of Chinese Medicine, Jiangsu Province Hospital of Chinese Medicine, The First Clinical Medical College, Nanjing, Jiangsu, China

**Keywords:** diabetic foot ulcers, transcriptomics, proteomics, metabolomics, multi-omics

## Abstract

**Background:**

Diabetes mellitus significantly increases the risk of complications, particularly diabetic foot ulcers (DFUs). However, the underlying mechanism remains unclear. This study aimed to assess the overall therapeutic approach in diabetic ulcers.

**Methods:**

Using integrated high-throughput multi-omics approaches, including transcriptomics, proteomics, and metabolomics, we constructed a compound-reaction-enzyme-gene network to identify the key molecular mechanisms involved in the pathogenesis of DFUs. Major findings were further validated in mouse models of diabetic and control ulcers.

**Results:**

Transcriptomics identified 653 differentially expressed genes (DEGs) between diabetic ulcers and control groups. Pathway analysis indicated that these genes were mostly related to inflammation, including the cytokine–cytokine receptor interaction, TNF signaling pathway, and NF-κB signaling pathway. Proteomics revealed 464 upregulated and 419 downregulated proteins, indicating many differentially expressed proteins (DEPs). The pathways with the highest representation of DEPs included diabetic cardiomyopathy, PPAR signaling pathway, and HIF-1 signaling pathway. Metabolomics identified 1,304 metabolites, predominantly lipids (32.1%) and organic acids (20.2%). Principal component analysis and partial least squares discriminant analysis confirmed the model’s effectiveness in distinguishing sample groups, whereas bioinformatics analysis revealed significant metabolic pathways, particularly amino acid biosynthesis.

**Conclusion:**

Our findings identified critical molecular signatures associated with DFUs and lay the groundwork for developing innovative therapeutic strategies to improve clinical outcomes in patients with this challenging condition.

## 1 Introduction

Diabetes mellitus (DM) arises from a complex interplay of genetic predisposition, immune dysregulation, infections, lifestyle, and psychosocial factors, leading to insulin resistance or pancreatic dysfunction. Currently, the global prevalence of diabetes among adults aged 20–79 years is estimated at 8.8%, with projections suggesting a potential increase to 693 million cases by 2045 if current trends persist (1). DFUs are significant complications associated with two chronic conditions of diabetes: peripheral neuropathy (PN) and peripheral arterial disease (PAD). Foot ulcers are a prominent manifestation of DFUs (2). DFUs affect up to 15% of individuals with diabetes and are the leading causes of hospitalization and both minor and major amputations in this population (2, 3).

Patients with DFUs often encounter precarious situations because foot ulcers are merely one aspect of a complex clinical picture. This intricacy includes persistent complications such as PN and PAD, compounded by comorbidities that collectively undermine the patient’s overall well-being. Notably, the 5-year mortality rate for individuals who develop new DFUs has been reported to surpass that of several cancers by approximately 25–60% (4, 5). Cardiovascular and renal diseases are leading contributors to mortality in this population. Patients with diabetic feet exhibit a complex interplay of inflammatory markers that adversely affects the cardiovascular system, thereby exacerbating cardiovascular damage and increasing morbidity. Consequently, effective management of DFU requires timely treatment of foot ulcers and a thorough assessment of comorbidities that may influence clinical outcomes (2, 6). However, the underlying mechanisms and potential targets of DFUs require further investigation.

High-throughput multi-omics techniques, including transcriptomics, proteomics, and metabolomics, hold promise for uncovering novel pathological mechanisms and identifying potential therapeutic targets. Transcriptomics enables qualitative and quantitative assessment of mRNA levels across the genome (7). In contrast, proteomics centers on elucidating alterations in protein expression under specific conditions and monitoring dynamic fluctuations within cellular environments (8). Metabolomics entails the comprehensive analysis of endogenous small biomolecules, primarily reflecting the end products of physiological processes mediated by proteins (9). However, a singular omics approach may fail to capture the full spectrum of changes associated with DFUs, given the dynamic and multifaceted nature of the condition. Independent analyses of extensive high-quality data across various omics levels often overlook the intricate interactions between different molecular entities, potentially missing critical biological insights (10). Therefore, a holistic strategy integrating data from multi-omics platforms is imperative for a comprehensive understanding of key pathological processes. Such integration can reveal new perspectives on complex biological systems and clarify networks of interactions at the molecular level.

In this study, we developed a comprehensive strategy to investigate diabetic ulcers, culminating in establishing a compound-reaction-enzyme-gene network. This network was constructed by integrating transcriptomic, proteomic, and metabolomic data to identify key targets and mechanisms for diabetic ulcer treatment. This approach may facilitate a deeper understanding of the molecular mechanisms underlying diabetic ulcers. Our findings present a detailed molecular map of these ulcers, advancing knowledge of their pathogenesis, and paving the way for exploring novel therapeutic interventions for affected patients.

## 2 Experimental methodologies

### 2.1 Clinical research

#### 2.1.1 Data sources and study population

**Figure 1:**
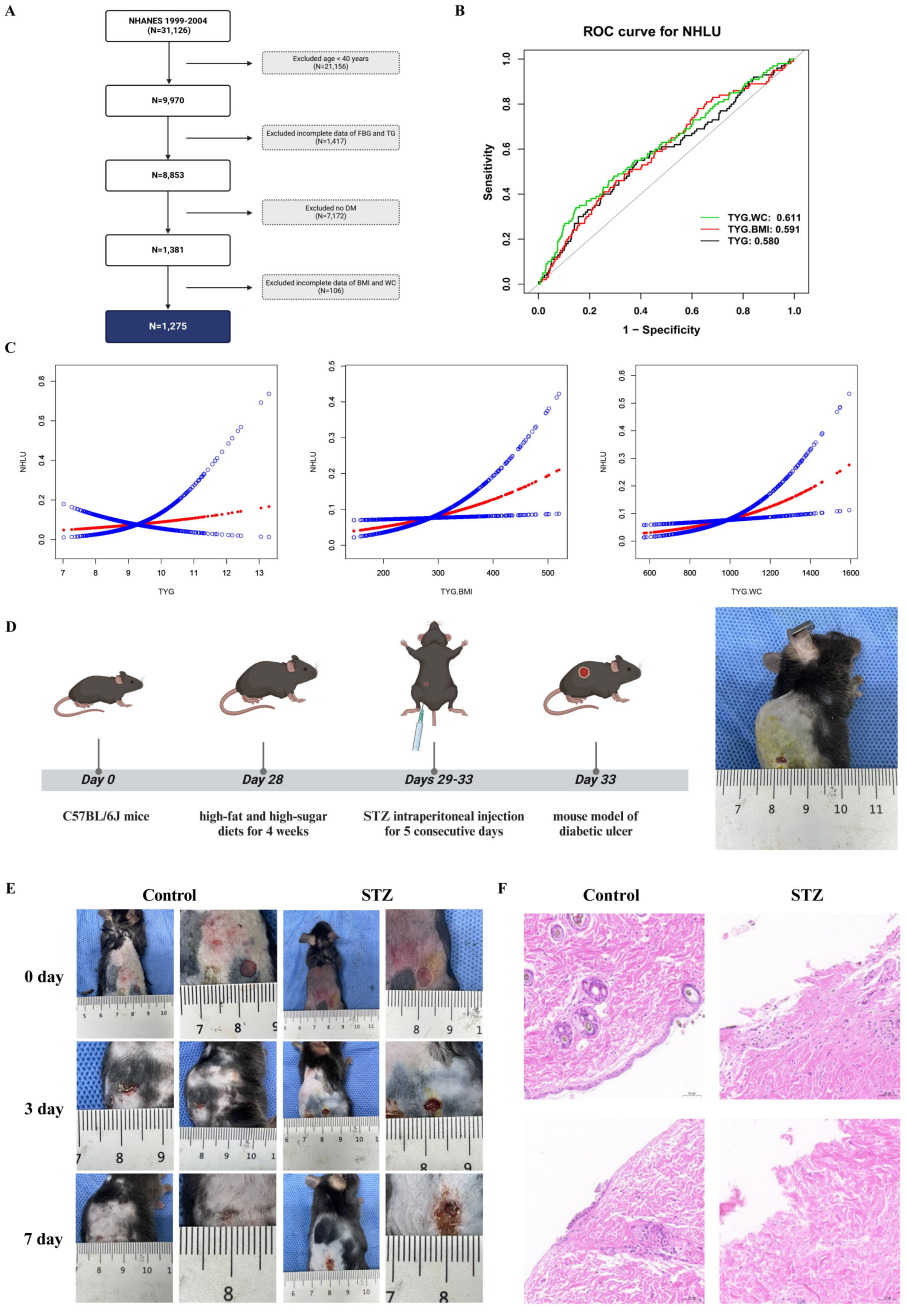
**(A)** Flowchart illustrating the methodology of this study. **(B)** Receiver Operating Characteristic (ROC) curves depicting the predictive utility of the TyG index, TyG-WC, and TyG-BMI for all-cause mortality in NHLU. **(C)** Smooth curve fitting analysis, with the solid red line representing the fitted curve between variables, and the blue bands indicating the 95% confidence interval surrounding this fit. **(D)** Development of a mouse model to study diabetic ulcers (this figure is created by biorender) and the representative wound healing photographs. **(E)** Representative images of ulcerated wounds across three groups of mice. **(F)** Assessment of dermal thickness among the three groups of mice using hematoxylin and eosin (HE) staining, scale bar = 50 μm.

The National Health and Nutrition Examination Survey (NHANES) comprises a series of cross-sectional, population-based studies aimed at evaluating the health and nutritional status of adults and children in the United States. The National Center for Health Statistics Research Ethics Review Board approved the NHANES study protocol, and all participants provided written informed consent. In accordance with the guidelines issued by the National Center for Health Statistics (NCHS), secondary use of NHANES data does not require additional IRB approval. This cross-sectional analysis utilized NHANES data (www.cdc.gov/nchs/nhanes/) collected between 1999 and 2004. A flowchart of the study design is shown in **Figure 1A**. Exclusion criteria included: (1) age under 40 years; (2) missing data on triglycerides, fasting glucose, body mass index (BMI), and waist circumference (WC); and (3) no DM diagnosis. Ultimately, 31,126 participants were enrolled, with 1,275 meeting the inclusion criteria. Among these, 100 participants (7.8%) were diagnosed with non-healing lower extremity ulcers (NHLU).

#### 2.1.2 Predictor and outcome variables

The primary outcome variable for this study was the presence of NHLU, defined by an affirmative response to the following question: “Have you had an ulcer or sore on your leg or foot that took more than four weeks to heal?” In recent years, studies have shown that triglyceride-glucose (TyG) index could be used as a reliable marker of insulin resistance (11, 12). Meanwhile, some reports have shown that TyG is associated with stroke and early diabetic nephropathy and is a reliable predictor of cardiovascular and all-cause mortality in prediabetes (13–15). The TyG index and its variants, TyG-WC and TyG-BMI, were calculated as follows: (1) TyG = ln [triglycerides (mg/dL) × glucose (mg/dL)/2]; (2) TyG-BMI = TyG × BMI; and (3) TyG-WC = TyG × WC. Additional covariates examined included age, sex, glycosylated hemoglobin, total cholesterol (TC), HDL, LDL, C-reactive protein (CRP), BMI, WC, systolic blood pressure (SBP), diastolic blood pressure, smoking history, and alcohol consumption.

### 2.2 Animals and regents

Twenty healthy male C57BL/6J mice (age: 8–10 weeks; body weight: 30–35 g) were purchased from the Beijing Viton Lever Company and housed in the Medical Research Center animal facility at the First Affiliated Hospital of Shandong First Medical University. The mice were divided into three cages, with five mice per cage. Two cages received a high-sugar, high-fat diet comprising 66.5% rat and mouse maintenance chow, 10% lard, 20% sucrose, 25% cholesterol, and 1% sodium cholate, while one cage received standard chow. Experiments commenced after a 4-week acclimatization period. All procedures involving mice followed the ARRIVE guidelines established by the Animal Protection Society of the First Affiliated Hospital of Shandong First Medical University **(Approval no. SYDWLS【2021】002)**.

### 2.3 Establishment of the diabetic ulcer mouse model

Ten C57BL/6J mice were fed a high-fat, high-sugar diet for 4 weeks, followed by a 12-h fasting period. They were then intraperitoneally injected with 2% streptozotocin (STZ) solution at 35 mg/kg for five consecutive days. Three days post-injection, blood glucose levels were measured in the tail vein to confirm the establishment of the diabetic mouse model. Subsequently, an oral glucose tolerance test was conducted to verify successful diabetes induction. Following the establishment of diabetes, hyperglycemia was maintained for an additional 2 weeks.

A skin ulcer model was created using ten diabetic and five normal mice. Anesthesia was induced through an intraperitoneal injection of 1% sodium pentobarbital, after which the mice were depilated and disinfected. Two points were marked on each mouse’s back, approximately 7 mm from the midline and 4 cm from the base of the neck, serving as the centers for wound creation. A sterile, disposable 5 mm biopsy punch was used to delineate the circular wound, and iris scissors (elbow type) were used to excise the tissue, creating an ulcer. The duration of wound induction was designated as 0 h. Afterward, the mice were housed individually, and photographs were taken and recorded on days 0, 3, and 7 (**Figures 1E, F**). Ulcer size in each group was observed, and the wound healing rate was calculated using ImagePro Plus 6.0, with the formula: wound healing rate (%) = [(initial wound area - wound area on the observation day)/initial wound area] × 100%. The diabetic ulcer mouse model and the representative wound healing photographs are illustrated in **Figure 1D**.

### 2.4 Multi-omics experimental phase investigation

#### 2.4.1 Transcriptomic analysis through RNA sequencing

To investigate transcriptional changes, high-throughput RNA sequencing was performed using the Illumina NovaSeq 6000 platform with 150 bp paired-end reads. Total RNA was extracted and subjected to quality assessment for purity and integrity using the NanoDrop 2000 (Thermo Fisher Scientific, USA) and LabChip GX Touch (PerkinElmer, USA). Ribosomal RNA (rRNA) was depleted using the Ribo-Zero Gold Kit (Illumina, USA) to enrich for non-rRNA transcripts. Strand-specific cDNA libraries were then constructed using the NEBNext Ultra Directional RNA Library Prep Kit (NEB, USA) following the manufacturer’s protocol. The library preparation workflow included RNA fragmentation, end repair, adapter ligation, size selection, and PCR enrichment.

The quality and concentration of the libraries were further evaluated using Qubit 3.0 (Invitrogen, USA), Agilent 2100 Bioanalyzer (Agilent Technologies, USA), and Bio-RAD CFX96 system (Bio-Rad, USA). Libraries were pooled based on effective concentration and sequencing depth requirements before being subjected to sequencing, which employed the sequencing-by-synthesis principle to generate high-quality reads. Raw sequencing data were initially processed using FastQC and Trim Galore to remove adapters, ambiguous reads (N bases), and low-quality sequences (Qphred ≤ 20 over more than 50% of the read length). Clean reads were aligned to the human reference genome (GRCh38) using HISAT2 (v2.2.1). Transcript assembly and quantification were performed with StringTie, and gene expression levels were calculated using both FPKM (Fragments Per Kilobase of transcript per Million mapped reads) and TPM (Transcripts Per Million).

Differential expression analysis was performed using DESeq2 for samples with biological replicates, applying negative binomial distribution models and adjusting *P*-values through the Benjamini–Hochberg method. Genes with adjusted *P*-values ≤ 0.05 were classified as differentially expressed. DESeq was used for samples without replicates. Finally, differential gene enrichment analysis was conducted using ClusterProfiler to identify significant Gene Ontology (GO) terms and KEGG pathways, applying a threshold of *P*-values < 0.05. The transcriptomic data analysis process is illustrated in **Supplementary Figure S1A**.

#### 2.4.2 Proteomic analysis

Protein extraction and digestion began with sample lysis using SDT buffer (4% (w/v) SDS, 100 mM Tris/HCl, pH 7.6, 0.1 M DTT), followed by protein quantification using a BCA Protein Assay Kit (Bio-Rad, USA). Proteins were digested according to the filter-aided sample preparation method described by Mann (16). Specifically, 200 μg of protein was treated with UA buffer (8 M Urea, 150 mM Tris-HCl, pH 8.0) to eliminate low-molecular-weight components using Microcon units (10 kD). This was succeeded by treatment with 100 μL of iodoacetamide (100 mM IAA in UA buffer) to block cysteine residues. An overnight trypsin digestion was performed, and the resulting peptides were desalted using Empore™ SPE Cartridges C18 (standard density, bed I.D. 7 mm, volume 3 mL, Sigma), concentrated, and reconstituted in 40 µL of 0.1% (v/v) formic acid. For SDS-PAGE analysis, 20 μg of each sample was combined with 5X loading buffer and boiled for 5 min.

Proteins were resolved on a 12.5% SDS-PAGE gel, with a constant current of 14 mA for 90 min, and visualized using Coomassie Blue R-250 staining. Peptides were then labeled with tandem mass tag (TMT) reagents (Thermo Scientific) and subjected to high-pH reversed-phase fractionation using a kit from Thermo Scientific, resulting in 10 separate peptide fractions. If there are too many samples, a bridge will be used for correction to reduce the batch effect. These fractions were desalted on C18 cartridges (Empore SPE Cartridges C18, standard density, bed I.D. 7 mm, volume 3 mL, Sigma) and concentrated by vacuum centrifugation. Liquid chromatography-tandem mass spectrometry (LC-MS/MS) analysis was performed using a Q Exactive mass spectrometer (Thermo Scientific), with a linear gradient of acetonitrile for peptide separation and data acquired in a data-dependent manner for identification and quantification. Raw MS data were processed using the MASCOT engine (version 2.2; Matrix Science, London, UK) in Proteome Discoverer 1.4 for protein identification and quantification.

After completing these steps, we conducted bioinformatics analysis to elucidate protein characteristics and interactions. Hierarchical clustering was performed using Cluster 3.0 (http://bonsai.hgc.jp/~mdehoon/software/cluster/software.htm) and Java Treeview software (http://jtreeview.sourceforge.net), with the Euclidean distance algorithm and average linkage clustering. The results were presented as heatmaps and dendrograms. Protein subcellular localization was predicted using the CELLO multiclass SVM classification system (http://cello.life.nctu.edu.tw/). Domain annotation was performed using InterProScan to identify the protein domain signatures from the Pfam database. GO annotations were derived using NCBI BLAST+ (ncbi-blast-2.2.28+-win32.exe) and InterProScan, with mapping and visualization achieved using Blast2GO and R scripts. KEGG annotations were obtained by blasting proteins against the KEGG database (http://geneontology.org/) to identify orthologs and associated pathways. Enrichment analyses were conducted using Fisher’s exact test and the Benjamini–Hochberg correction for multiple testing, highlighting functional categories and pathways with *P*-values < 0.05. Finally, protein–protein interactions were explored using the IntAct database (17) (http://www.ebi.ac.uk/intact/) and STRING (http://string-db.org/), with networks visualized in Cytoscape (http://www.cytoscape.org/, version 3.2.1) to assess the significance of each protein within the interaction network based on degree. The proteomic analysis is illustrated in **Supplementary Figure S1B**.

#### 2.4.3 Metabolomic analysis

**Chemicals:** Ammonium acetate was procured from Sigma Aldrich, acetonitrile from Merck, and ammonium hydroxide and methanol from Fisher Scientific. After dissection, mouse tissues were rapidly frozen in liquid nitrogen. Approximately 80 mg of tissue was diced on dry ice, placed in a 2 mL Eppendorf tube, and homogenized with a mixture of 200 μL H2O and five ceramic beads using a homogenizer. For metabolite extraction, 800 μL of methanol/acetonitrile (1:1, v/v) solution was added to the homogenate. The mixture was centrifuged at 14,000 g for 20 min at 4°C, and the supernatant was dried using a vacuum centrifuge. For LC-MS analysis, the dried samples were re-dissolved in 100 μL of a 1:1 (v/v) acetonitrile/water solution and centrifuged again at 14,000 g for 15 min at 4°C, and the supernatant was injected for analysis.

To ensure the stability and reproducibility of the instrument analysis, quality control samples were generated by pooling 10 μL from each sample. In this experiment, the number of peaks with RSD ≤ 30% in the QC samples accounted for more than 80% of the total number of peaks in the QC samples, indicating that the instrument analysis system has good stability and the data can be used for subsequent analysis. These quality control samples were incorporated into the analysis at regular intervals and evaluated after every five samples. Chromatography-mass spectrometry analysis was performed using a Vanquish ultra-high-performance liquid chromatography (UHPLC) system with a HILIC column (Vanquish UHPLC, Thermo Scientific), with samples processed randomly. A Q Exactive series mass spectrometer was used to collect both primary and secondary spectra. The raw data were then converted to mzXML format using ProteoWizard MSConvert. Peak alignment, retention time correction, and peak area extraction were conducted using XCMS. The data extracted by XCMS were subjected to metabolite structure identification and pre-processing, followed by quality evaluation and subsequent analyses.

After sum normalization, data were analyzed using the R package (ropls) for multivariate analysis, including principal component analysis (PCA) and orthogonal partial least-squares discriminant analysis (OPLS-DA). Model robustness was evaluated through 7-fold cross-validation and response permutation testing. Variable importance in projection (VIP) values were calculated to determine each variable’s contribution to classification, with significant metabolites identified using VIP > 1 and *P* < 0.05. Student’s t-test was used to determine significance between independent sample groups, and Pearson’s correlation analysis was performed to explore the relationships between variables. The metabolomic analysis is illustrated in **Supplementary Figure S1C**.

### 2.5 Statistical analysis

In this study, continuous variables were presented as means with standard deviations and categorical variables as counts with their corresponding proportions. Differences between the two groups were analyzed using weighted linear regression for quantitative variables and weighted chi-squared tests for qualitative variables. We utilized multiple logistic regression models to estimate the association of TyG, TyG-BMI, and TyG-WC with NHLU, with the first group as a reference. We developed three multivariate models to further elucidate the clinical implications of the logistic regression findings. Model 1 incorporated TyG, TyG-BMI, and TyG-WC as predictors of NHLU. Model 2 was adjusted for age and sex, while Model 3 included additional covariates, including smoking status, alcohol consumption, fasting blood glucose (FBG), hemoglobin A1c (HbA1c), triglycerides (TG), TC, CRP, and SBP. Analyses were conducted using EmpowerStats (http://www.empowerstats.com/cn/) and R. All probability values were two-sided, with interaction *P*-values < 0.05 considered statistically significant.

## 3 Results

### 3.1 Clinical characteristics

This study enrolled a total of 1,275 patients with type 2 diabetes mellitus (T2DM), of whom 100 presented with NHLU. **Table 1** shows the clinical characteristics of the participants categorized by NHLU status. Significant differences were identified in WC, FBG, TyG index, TyG-WC, and TyG-BMI (all with *P* < 0.05). **Table 2** presents the prevalence of NHLU among patients with T2DM, stratified by quartiles of TyG index, TyG-WC, and TyG-BMI. The Q4 group demonstrated the highest prevalence of NHLU across all three indicators, with significant differences observed in each case (*P* < 0.05). Specifically, within the TyG-WC quartiles, NHLU prevalence was the highest in group Q4, followed by Q3, Q2, and Q1, all with significant differences (*P* < 0.05). For the TyG quartiles, NHLU prevalence was significantly higher in Q4 and Q3 than in Q2 and Q1 (*P* < 0.05). Additionally, among those grouped by TyG-BMI quartiles, Q4 exhibited a greater NHLU prevalence than Q1, with significant differences observed between the groups (*P* < 0.05).

**Table 1:** Clinical characteristics of participants with and without non-healing lower extremity ulcers (NHLU).

Characteristics	Non-NHLU (n=1175)	NHLU (n=100)	*P* value
Age (years)	63.98 ± 11.09	63.96 ± 11.12	0.98
HbA1c (%)	7.42 ± 1.80	7.37 ± 1.80	0.705
FBG, mg/dL	145.16 ± 72.21	173.88 ± 99.96	<0.001
HDL-C, mg/dL	48.19 ± 13.62	48.02 ± 16.45	0.905
LDL-C, mg/dL	114.88 ± 36.58	105.09 ± 30.02	0.134
TC, mg/dL	201.81 ± 46.41	198.31 ± 43.61	0.468
CRP, mg/dL	0.68 ± 1.53	0.67 ± 0.79	0.933
TG, mg/dL	202.74 ± 220.93	220.98 ± 188.51	0.423
SBP, mmHg	137.59 ± 22.16	138.41 ± 22.35	0.738
DBP, mmHg	68.14 ± 16.64	67.72 ± 19.51	0.824
BMI, kg/m2	30.70 ± 6.34	32.00 ± 6.90	0.050
WC (cm)	105.69 ± 14.16	110.24 ± 15.61	0.002
TyG index	9.27 ± 0.83	9.51 ± 0.89	0.005
TyG.BMI	284.91 ± 65.00	303.66 ± 67.32	0.006
TyG.WC	980.90 ± 162.5	1048.84 ± 176.85	<0.001
Gender (%)			0.177
Male	599 (50.98%)	58 (58.00%)	
Female	576 (49.02%)	42 (42.00%)	
Smoking (%)			0.882
No	982 (83.57%)	83 (83.00%)	
Yes	193 (16.43%)	17 (17.00%)	
Alcohol drinking (%)			0.059
No	980 (83.40%)	76 (76.00%)	
Yes	195 (16.60%)	24 (24.00%)	

TyG, Triglyceride-Glucose; TG, Triglyceride; WC, waist circumference; FBG, fasting blood glucose; TyG-WC, Triglyceride glucose-waist circumference; TyG-BMI, Triglyceride glucose-body mass index; CRP, C-reactive protein; SBP, Systolic blood pressure; DBP, Diastolic blood pressure; BMI, Body mass index.

**Table 2:** Clinical characteristics of participants with and without NHLU.

Characteristics	Non-NHLU (n=1175)	NHLU (n=100)	*P* value
TyG index			0.045
Q1	298 (25.36%)	21 (21.00%)	
Q2	300 (25.53%)	18 (18.00%)	
Q3	294 (25.02%)	25 (25.00%)	
Q4	283 (24.09%)	36 (36.00%)	
TyG.BMI			0.010
Q1	303 (25.79%)	16 (16.00%)	
Q2	294 (25.02%)	24 (24.00%)	
Q3	297 (25.28%)	22 (22.00%)	
Q4	281 (23.91%)	38 (38.00%)	
TyG.WC			0.006
Q1	302 (25.70%)	17 (17.00%)	
Q2	298 (25.36%)	20 (20.00%)	
Q3	295 (25.11%)	24 (24.00%)	
Q4	280 (23.83%)	39 (39.00%)	

TyG, Triglyceride-Glucose; TyG-WC, Triglyceride glucose-waist circumference; TyG-BMI, Triglyceride glucose-body mass index.

### 3.2 Multivariate analysis of determinants of NHLU in study participants

A multivariate logistic regression model was constructed to investigate the associations between the TyG index, TyG-WC, TyG-BMI, and NHLU in patients with DM, as outlined in **Table 3**. In Model 1, no adjustments were made for confounding variables. Elevated levels of the TyG index, TyG-WC, and TyG-BMI (group Q4) exhibited significant positive correlations with NHLU when compared to group Q1, yielding odds ratios of 0.59 (95% CI: 0.02–1.17; *P* = 0.0440), 0.91 (95% CI: 0.33–1.49; *P* = 0.0022), and 0.94 (95% CI: 0.33–1.55; P = 0.0023), respectively. In Model 2, adjusted for age and sex, the associations remained significant. The highest TyG index, TyG-WC, and TyG-BMI levels in group Q4 remained positively associated with NHLU compared to the reference group, with odds ratios of 0.60 (95% CI: 0.02–1.19; *P* = 0.0441), 1.03 (95% CI: 0.40–1.65; *P* = 0.0013), and 1.19 (95% CI: 0.53–1.86; *P* = 0.0004), respectively. In Model 3, adjusted for age, sex, smoking status, alcohol consumption, FBG, HbA1c, TG, TC, CRP, and SBP, the highest TyG-WC and TyG-BMI levels remained positively associated with NHLU, with odds ratios of 0.93 (95% CI: 0.10–1.77; *P* = 0.0289) and 1.25 (95% CI: 0.34–2.15), respectively. However, no significant relationship was observed between the TyG index and NHLU.

**Table 3:** Logistic regression analysis of different indexes on the risk of NHLU in T2DM patients.

Indicators	Model 1 β (95% CI) P-value	Model 2 β (95% CI) P-value	Model 3 β (95% CI) P-value
TyG index			
Q1 (7.02 - 8.72)	Reference	Reference	Reference
Q2 (8.72 - 9.20)	-0.16 (-0.83, 0.51) 0.64	-0.24 (-0.93, 0.46) 0.51	-0.47 (-1.36, 0.42) 0.31
Q3 (9.20 - 9.78)	0.19 (-0.42, 0.80) 0.55	0.16 (-0.49, 0.80) 0.64	-0.13 (-0.93, 0.68) 0.76
Q4 (9.78 - 13.30)	0.59 (0.02, 1.17) 0.04	0.60 (0.02, 1.19) 0.04	0.16 (-0.89, 1.21) 0.76
TyG-BMI			
Q1 (139.50 - 240.46)	Reference	Reference	Reference
Q2 (240.60 - 275.63)	0.44 (-0.17, 1.04) 0.16	0.61 (-0.06, 1.27) 0.07	1.06 (0.24, 1.89) 0.01
Q3 (275.73 - 322.64)	0.34 (-0.31, 0.99) 0.31	0.47 (-0.23, 1.18) 0.18	0.48 (-0.46, 1.43) 0.32
Q4 (323.17 - 602.22)	0.94 (0.33, 1.55) 0.002	1.19 (0.53, 1.86) 0.001	1.25 (0.34, 2.15) 0.007
TyG-WC			
Q1 (525.91 - 874.46)	Reference	Reference	Reference
Q2 (874.61 - 972.06)	0.18 (-0.48, 0.84) 0.60	0.35 (-0.35, 1.04) 0.33	0.46 (-0.38, 1.30) 0.29
Q3 (972.43 - 1086.67)	0.37 (-0.25, 0.98) 0.24	0.44 (-0.24, 1.12) 0.20	0.36 (-0.48, 1.19) 0.40
Q4 (1087.22-1616.15)	0.91 (0.33, 1.49) 0.002	1.03 (0.40, 1.65) 0.001	0.93 (0.10, 1.77) 0.029

Model 1: Unadjusted; Model 2: Adjusted for age and gender; Model 3: Adjusted for age, gender, smoking, alcohol consumption, FBG, HbA1c, Triglyceride (TG), Total cholesterol (TC), C-reactive protein (CRP), and SBP.

### 3.3 Predictive value of TyG-related indices for NHLU in patients with T2DM

To evaluate the predictive capacity of the TyG index, TyG-WC, and TyG-BMI for NHLU in patients with DM, we conducted a receiver operating characteristic (ROC) curve analysis (**Figure 1B**; **Table 4**). The identified cutoff values were as follows: TyG index at 9.43 (sensitivity, 55%; specificity, 61%; area under the curve [AUC], 0.58), TyG-WC at 1150.80 (sensitivity, 34%; specificity, 85%; AUC, 0.61), and TyG-BMI at 307.22 (sensitivity, 46%; specificity, 70%; AUC, 0.59). The predictive model showed an overall sensitivity of 44.6% and an AUC of 0.607.

**Table 4:** Ability of TyG, TyG-BMI, TyG-WC to predict NHLU in DM patients.

Indicators	AUC (95%CI)	Best threshold	Specificity	Sensitivity
TyG index	0.58	9.43	0.61	0.55
TyG-BMI	0.59	307.22	0.70	0.46
TyG-WC	0.61	1150.80	0.85	0.34

### 3.4 Linear relationship between TyG-WC and TyG-BMI in DM and NHLU

We conducted a smoothed curve-fitting analysis to assess the relationship between TyG indices in DM and NHLU (**Figure 1C**). After adjusting for all covariates in Model 3, we identified a significant linear relationship between TyG-WC and TyG-BMI in the context of DM and NHLU (*P* < 0.05).

### 3.5 Transcriptomic analysis of the diabetic ulcers mice model

We assessed the expression levels of the samples using the formula:


$$FPKM=\frac{10^{3}*F}{NL/10^{6}}.$$

**Figure 2:**
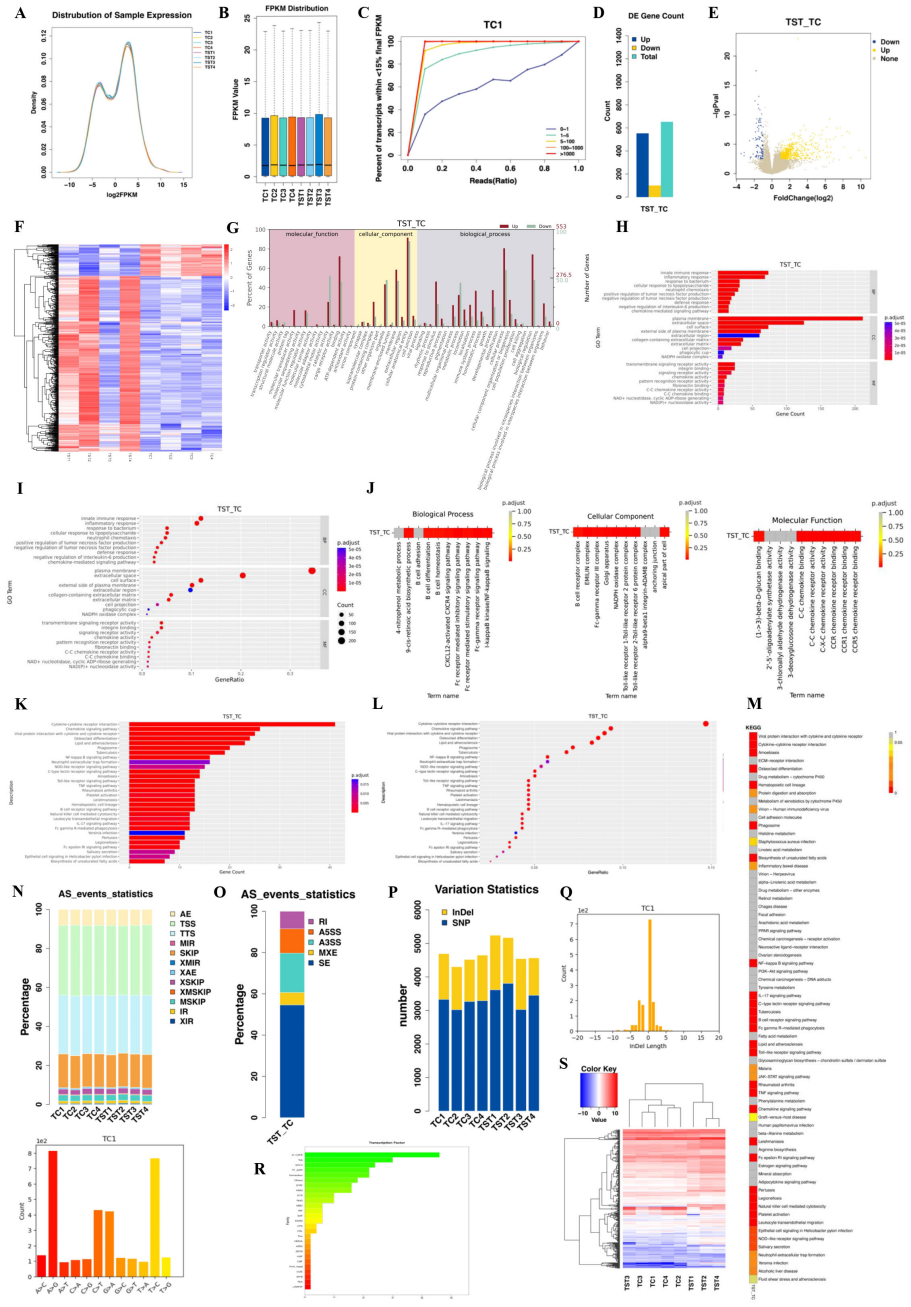
**(A)** Expression distribution diagram. The horizontal axis is log2(FPKM), which represents the logarithmic value of the gene expression level of each sample. The higher the value, the higher the gene expression level. The vertical axis is the ratio of the number of genes at the corresponding expression level to the total number of genes. **(B)** Box plot of expression level. The horizontal axis is the sample name, and the vertical axis is the logarithmic value of the gene expression of each sample. From top to bottom, they represent the maximum value, upper quartile, median, lower quartile and minimum value. **(C)** Saturation analysis chart. Different colors represent genes classified based on expression levels. The horizontal axis is the ratio of sampled reads to the total number of reads during random sampling, and the vertical axis represents the percentage of genes whose expression levels could be accurately estimated through sampled reads. **(D)** Statistical graph of differentially expressed genes. The horizontal axis represents different sets of differentially expressed genes, green represents all differentially expressed genes, blue represents upregulated genes, yellow represents downregulated genes, and the vertical axis represents the number of differentially expressed genes. **(E)** Volcano plot of DEGs. The horizontal axis represents the expression fold change of genes in different samples, and the vertical axis represents the statistical significance of the expression change. Blue represents significantly downregulated genes, yellow represents significantly upregulated genes, and gray represents genes with no significant change in expression. **(F)** Differential gene clustering diagram. Behavioral genes are listed as samples. Red indicates high gene expression, and blue indicates low gene expression. The horizontal axis represents the clustering of samples, and the vertical axis represents the clustering of genes. **(G)** GO statistical bar chart of DEGs. The horizontal axis is GO Term, the left vertical axis is the percentage of the number of genes, and the right vertical axis is the number of genes. **(H)** GO enrichment bar chart. The vertical axis represents the GO entry, the horizontal axis represents the number of genes enriched in the entry, and the color represents p.adjust. The redder the color, the more significant it is. **(I)** GO enrichment bubble chart. The bubble size indicates the number of genes enriched in this entry. The larger the bubble, the more genes there are. **(J)** Distribution of p.adjust values of enriched GO terms (including BP, CC and MF). **(K)** KEGG enrichment bar chart. **(L)** KEGG enrichment bubble chart. **(M)** Distribution of p.adjust values of enriched pathways. Different colors represent different degrees of enrichment; the redder the color, the more significant the enrichment. **(N)** Statistics of variable splicing results. The horizontal axis represents the sample name, and the vertical axis represents the percentage of alternative splicing event types. **(O)** Statistics of differential alternative splicing results. **(P)** Distribution diagram of mutation statistics. The horizontal axis represents the sample name, and the vertical axis represents the number of mutations. **(Q)** SNP mutation frequency distribution and InDel length distribution. A>C represents the number of SNP sites that mutated from A to C. **(R)** Distribution diagram of differential transcription factor protein families. The vertical axis represents different transcription factor families, and the horizontal axis represents the number of differentially expressed genes annotated to the transcription factor. **(S)** Cluster diagram of differential transcription factors. Red indicates high gene expression, and blue indicates low gene expression. The horizontal axis represents the clustering of samples, and the vertical axis represents the clustering of genes.

The resulting expression density map illustrated gene expression concentrations within the peak area (**Figure 2**). A box plot was generated to illustrate the expression levels across samples and revealed a consistent trend (**Figure 2**). Additionally, a saturation analysis confirmed that the data volume in this study was adequate for further analysis (**Figure 2**).

Differential gene screening identified 553 upregulated and 100 downregulated genes in the TC group compared with the TST group, totaling 653 differentially expressed genes (DEGs) (**Figure 2**). DEGs were defined as genes showing statistically significant differences in expression levels between two comparison groups, identified using the DESeq2 package with a threshold of |log_2_ fold change| > 1 and adjusted *p*-value < 0.05. A volcano plot was generated to visually represent the distribution of these DEGs across groups (**Figure 2**). To further elucidate gene expression differences and identify novel functional genes, hierarchical clustering analysis was performed on all screened DEGs. Genes exhibiting similar expression patterns across samples were clustered and visualized using heatmaps (**Figure 2**; **Data S12**). Following DEG identification, we conducted GO functional enrichment and KEGG pathway enrichment analyses to elucidate the fundamental molecular mechanisms underlying the biological processes involved. The GO statistical results, summarized in a bar graph (**Figure 2**), indicate that molecular functions were predominantly enriched in binding, cellular components were primarily associated with cellular, anatomical entities, and biological processes largely involved cellular processes and biological regulation. The 30 most significant GO terms were visualized in bar and bubble charts (**Figures 2H**; **Data S13**). Additionally, a distribution diagram illustrates the adjusted enrichment significance values across samples (**Figure 2**).

Subsequently, we identified the 30 most significant pathways (or all if fewer than 30) through KEGG enrichment analysis, with results indicating significant enrichment in pathways such as cytokine–cytokine receptor interaction, NF-κB signaling, and TNF signaling (**Figures 2K**; **Data S14**). A distribution map illustrates the adjusted *P*-values of the enriched pathways across all comparison groups (**Figure 2**).

Alternative splicing is a critical regulatory mechanism that contributes to gene expression diversity and is essential for growth and development. Using ASProfile software, we analyzed and quantified the variable splicing events per sample based on known gene models (**Figure 2**). Additionally, rMATS was used to classify and count alternative splicing events for each comparison group (**Figure 2**). We also investigated single nucleotide polymorphisms (SNPs), which represent allele mutations across the genome. Using SAMtools, we compared the alignment files, post-sorting and PCR duplication removal against the reference sequence to detect variations, revealing approximately 3,000 SNP mutations (**Figure 2**). We subsequently recorded the frequency and length of each mutation based on the identified SNP sites (**Figure 2**; **Supplementary Figure S2**).

Transcription initiation in eukaryotes is complex and often involves multiple protein factors. We predicted transcription factors and compared our findings with the Animal Transcription Factor Database (AnimalTFDB) to categorize these factors by family. The distribution of the predicted transcription factors mapped to their respective protein families is illustrated in **Figure 2**, with notable prevalence in the zf-C2H2 and TF_bZIP families. To reflect the expression patterns of the differentially expressed transcription factors under varying experimental conditions, we conducted a hierarchical cluster analysis using R. This analysis revealed that transcription factors within the same cluster exhibited similar expression trends under the same experimental conditions (**Figure 2**).

### 3.6 Proteomic analysis of diabetic ulcers mice model

**Figure 3:**
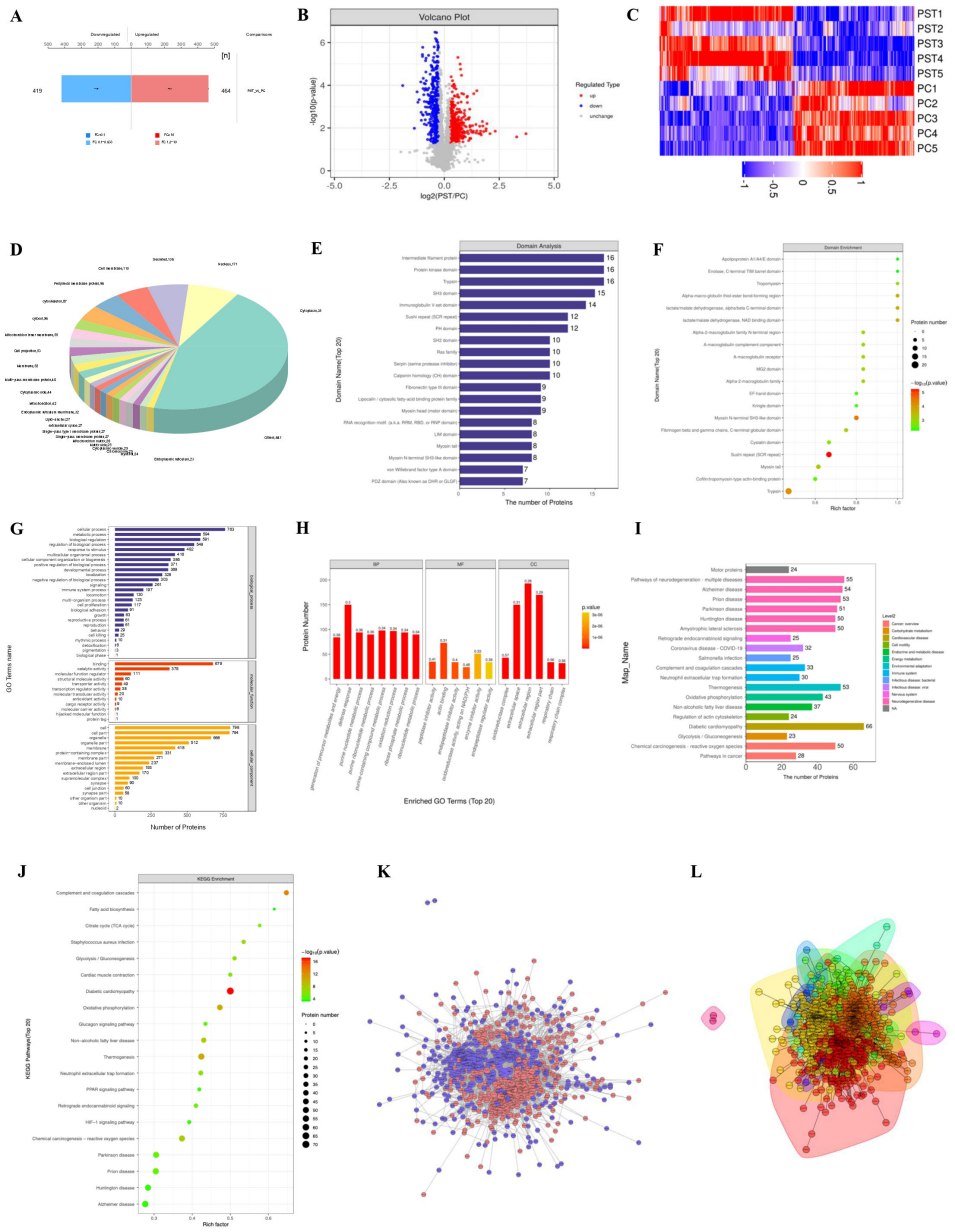
**(A)** Bar graph of protein quantification difference results. **(B) ** Volcano plot of the PST*vs*PC group. The horizontal axis is the difference fold (logarithmic transformation with base 2), and the vertical axis is the significant *P*-value of the difference (logarithmic transformation with base 10). The red points in the figure are upregulated proteins with significant differential expression, the blue points are downregulated proteins with significant differential expression, and the gray points are proteins with no difference. **(C)** PST*vs*PC group differentially expressed protein clustering analysis results. Hierarchical clustering results are presented in a tree-type heatmap, where each column represents a group of samples (the horizontal axis is sample information), and each row represents a protein (i.e., the vertical axis is the protein with significant differential expression). **(D)** Pie chart of subcellular localization of differentially expressed proteins in the PST*vs* PC group. **(E)** PST*vs*PC group differentially expressed protein domain analysis diagram. **(F)** PST*vs*PC group domain enrichment analysis. The horizontal axis in the figure is the enrichment factor (Rich Fator ≤ 1), which indicates the ratio of differentially expressed proteins annotated to the GO category to the number of all identified proteins annotated to the category. The vertical axis indicates the statistical results of differential proteins under each domain classification; the color of the bubble indicates the significance of the enriched domain classification. **(G)** GO annotation statistics of differentially expressed proteins in the PST*vs*PC group (level 2). **(H)** GO annotation statistics of the top 20 differentially expressed proteins in the PST*vs*PC group. **(I)** A diagram of biological metabolic processes showing the relationship between different metabolic processes and the relevant values. **(J)** KEGG pathway enrichment bubble diagram of PST*vs*PC group. **(K)** PST*vs*PC group differentially expressed protein interaction network diagram. **(L)** Interaction network diagram for functional classification.

Through proteomic analysis, we generated a total of 811,606 secondary spectra, which included 80,869 spectra matched to the database, 31,709 peptides (of which 28,252 were unique), and 4,985 identified proteins, of which 4,983 were quantifiable (**Supplementary Figure S3**; **Data S1** and **S2**). To investigate differential protein expression across groups, we applied a 1.2-fold change (FC) threshold at *P* < 0.05, identifying 883 differentially expressed proteins (DEPs) between the PC and PST groups. DEPs refer to proteins whose abundance significantly varies between experimental conditions, as determined from proteomic datasets using Student’s *t*-test or ANOVA, with FDR correction applied where appropriate. These DEPs included 464 upregulated and 419 downregulated proteins (**Figure 3**; **Data S3**).

To identify significant protein differences, we generated a volcano plot, displaying FC and *P-*values from t-tests. Proteins exhibiting substantial downregulation are indicated in blue (FC < 0.83, *P* < 0.05), whereas significantly upregulated proteins are indicated in red (FC > 1.2, *P* < 0.05). Proteins without significant differences are represented in gray (**Figure 3**). Subsequent cluster analysis revealed distinct differences in protein expression between the PC and PST groups (**Figure 3**).

Subcellular localization of all DEPs was analyzed using the CELLO subcellular structure prediction software (18). The results indicated that most DEPs were localized in the cytoplasm (356 proteins, 46%), followed by the nucleus (171 proteins, 22%) (**Figure 3**; **Data S4** **).** To predict DEP domains, we utilized InterProScan to identify the top 20 protein domains, with the most prominent being intermediate filament proteins, protein kinase domains, and trypsins (**Figure 3** **).** To elucidate the domain enrichment characteristics of the DEPs, we conducted domain enrichment analysis using Fisher’s exact test, revealing significant enrichment in sushi repeat (SCR repeat) and trypsin domains (**Figure 3**).

To comprehensively understand the function, localization, and biological pathways of the proteins, we performed GO functional annotation using Blast2Go (https://www.blast2go.com/) and assessed secondary functional annotation levels (**Figure 3**). Fisher’s exact test was applied to identify enriched functional categories among DEPs, revealing significant alterations in key biological processes, including generating precursor metabolites and energy, defense responses, and purine nucleotide metabolism. Additionally, significant changes were observed in molecular functions, including peptidase and endopeptidase inhibitor activities, actin binding, and localization within components like the oxidoreductase complex and extracellular space (**Figure 3**; **Data S5**).

To illustrate the hierarchical relationships among GO terms associated with DEPs, we employed a topGO-directed acyclic graph. This top-down visualization presents the functional scope, with branches reflecting inclusion relationships and lower branches corresponding to more specific functional categories (**Supplementary Figure S4**). The proteins were systematically annotated using the KEGG pathway database (**Figure 3**), tallying the DEPs associated with each pathway, and revealing diabetic cardiomyopathy (DCM), the PPAR signaling pathway, and the HIF-1 signaling pathway as the most prominent pathways (**Figure 3**; **Data S6**). Visual representations of the PPAR signaling pathway and HIF-1 signaling pathway are shown in **Supplementary Figure S5**.

Given that highly aggregated proteins may share similar functions and that highly connected proteins can act as pivotal nodes influencing metabolic or signaling pathways, we further investigated protein–protein interactions. The resulting interaction network diagram of DEPs in the PST vs. PC group, along with specific clusters, is presented in **Figures 3, L**.

### 3.7 Metabolomic analysis of diabetic ulcers mice model

**Figure 4:**
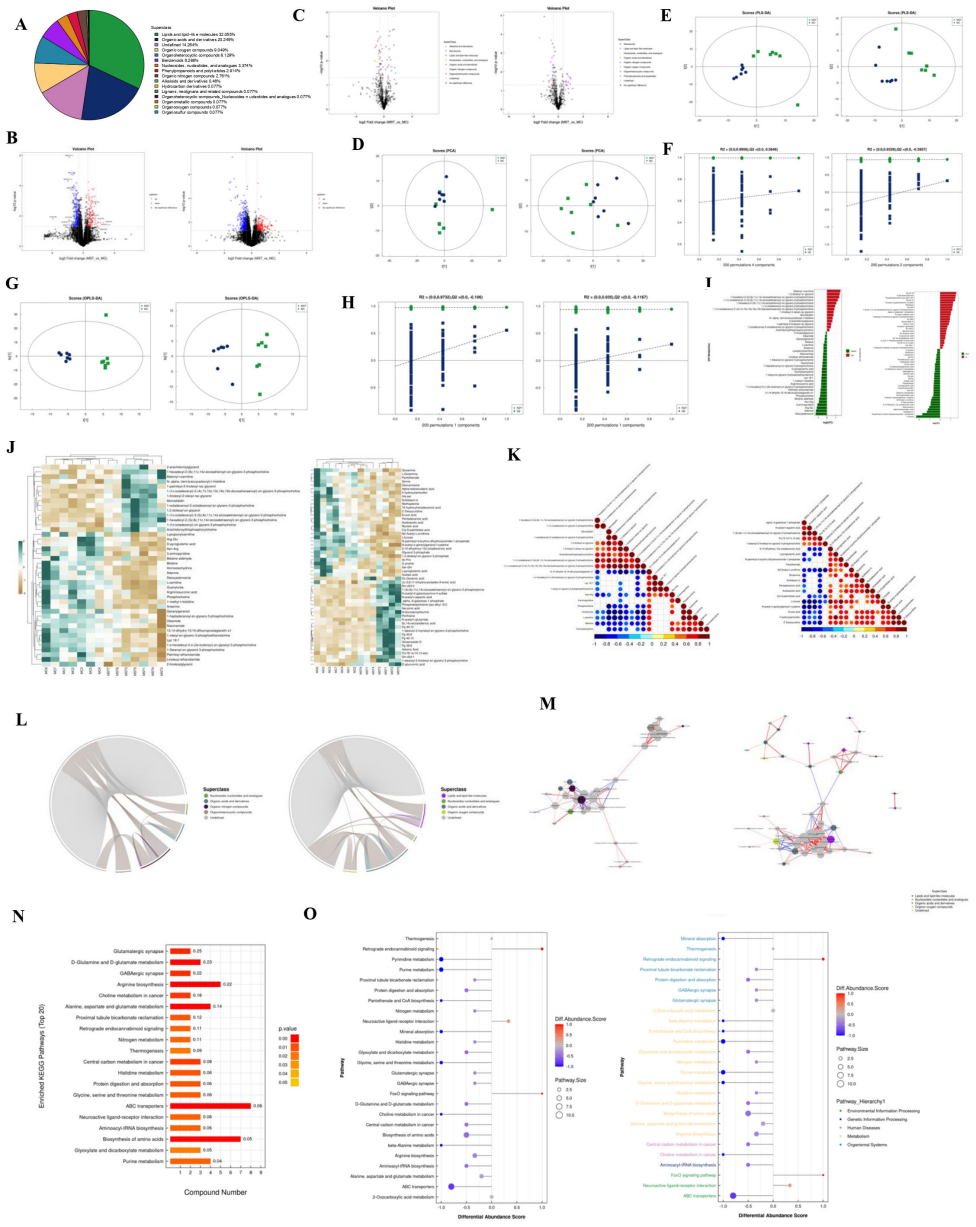
**(A)** The proportion of identified metabolites in each chemical category. The blocks of different colors in the figure represent different chemical classification entries, and the percentage represents the percentage of metabolites in the chemical classification entry to the total number of identified metabolites. Metabolites without chemical classification are defined as undefined. **(B)** Volcano plots of positive and negative ion modes. Upregulated metabolites are represented by rose red, downregulated metabolites are represented by blue, and non-significantly different metabolites are represented by black. **(C)** Negative ion mode volcano plot (colors are related to the chemical classification of differential metabolites). **(D)** PCA score plots for positive and negative ion modes. t [1] represents principal component 1, t [2] represents principal component 2, and the ellipse represents the 95% confidence interval. Points of the same color represent the biological replicates within the group, and point distribution reflects the differences between and within the groups. **(E)** PLS-DA score plots for positive and negative ion modes. **(F)** PLS-DA permutation test in positive and negative ion modes. **(G)** OPLS-DA score plots for positive and negative ion modes. **(H)** OPLS-DA permutation test in positive and negative ion modes. **(I)** Analysis of fold difference in expressing significant differential metabolites in positive and negative ion modes. **(J)** Hierarchical clustering heat map of significantly different metabolites in positive and negative ion modes. Each row in the figure represents a differential metabolite (i.e., the vertical axis is the metabolite with significant differential expression), and each column represents a group of samples (i.e., the horizontal axis is the sample information). **(K)** Positive and negative ion mode correlation heatmap. Red indicates a positive correlation, blue indicates a negative correlation, and white indicates a non-significant correlation. **(L)** Positive and negative ion mode chord diagram. The starting point of the inner circle link in the figure represents each significantly different metabolite, and the arc on the outer circle represents the category to which the significantly different metabolites belong. The colored lines represent the correlation within each metabolite, and the lines are the same color as the subclasses. The dark gray lines represent the correlation between metabolites of different categories. **(M)** Positive and negative ion mode network diagram. The dots in the figure represent significantly different metabolites. The size of the dots is related to the degree of connectivity. The larger the degree, the larger the dots. The color of the line represents the correlation. Red indicates positive correlation, and blue indicates negative correlation. The thickness of the line represents the absolute value of the correlation coefficient. The thicker the line, the greater the correlation. **(N)** KEGG enrichment pathway diagram (bar graph). The vertical axis in the bar graph represents each KEGG metabolic pathway, and the horizontal axis represents the number of differentially expressed metabolites contained in each KEGG metabolic pathway. **(O)** The differential abundance score map of all enriched metabolic pathways and the score map after classification according to Pathway_Hierarchy.

For metabolite identification, we used an in-house database (19, 20) to match retention times, molecular masses (error margin < 10 ppm), secondary fragmentation spectra, and collision energy data from biological samples. A rigorous manual validation confirmed that all identifications achieved a minimum Level 2 classification. After integrating positive and negative ion modes, 1,304 metabolites were identified, with the breakdown of metabolites detected in each mode presented in **Table 5** and detailed qualitative and quantitative results in **Data S7**. Metabolites were categorized by their chemical taxonomy, revealing lipids and lipid-like molecules as the largest proportion (32.055%), followed by organic acids and their derivatives (20.245%) (**Figure 4**).

**Table 5:** Statistics of metabolites identified in positive and negative ion modes.

Detection mode	Number of metabolites identified
Positive ion mode (Pos)	680
Negative ion mode (Neg)	624

Metabolomic data, characterized by high dimensionality and strong intervariable correlations, were analyzed using multivariate statistical methods to integrate univariate analyses with multidimensional assessments, identifying group differences and potential biomarkers. Differential analysis included all metabolites detected in both ion modes and those that remained unidentified, visualized as volcano plots with significant changes indicated by FC > 1.5 or < 0.67 and *P* < 0.05 (**Figure 4** **).** We used distinct colors to visually represent the classification of these differential metabolites, as depicted in **Figure 4**.

PCA, an unsupervised method, was employed to linearly combine all identified metabolites into a new set of variables. This method aims to reflect as much information as possible from the original variables while achieving dimensionality reduction. PCA results provided insights into overall distribution trends and inter-group differences (**Figure 4**), with model parameters validated through a seven-fold cross-validation (**Data S8a; b**), indicating an R² value approaching 1, confirming the reliability of our findings. Subsequently, we established a discriminant model using Partial Least Squares Discriminant Analysis (PLS-DA) to identify the differential lipid substances associated with the groups (**Figure 4**). The PLS-DA model effectively separated the two sample groups, with robust stability confirmed by evaluation parameters (R²Y, Q²) (**Data S8c, d**). To mitigate the risk of overfitting in the supervised model, we performed a permutation test (**Figure 4**). The decreasing R² and Q² values of the random models confirmed the absence of overfitting and the model’s robustness.

To enhance the rigor of our analysis, we applied OPLS-DA, which further distinguished the two sample groups. Circular cross-validation confirmed model stability and reliability (**Figure 4**; **Data S8e, f**), and a permutation test validated the absence of overfitting (**Figure 4**). Following common metabolomic practices, we employed stringent criteria (VIP > 1 and *P* < 0.05) to screen for significantly differential metabolites (**Figure 4**; **Data S9**). Subsequently, we performed bioinformatics analyses, including cluster, correlation, and pathway analyses, on these metabolites.

To comprehensively illustrate sample relationships and metabolite expression patterns, we clustered and analyzed the expression levels of all samples with the differential metabolites (**Figure 4** **).** Correlation analysis assessed the metabolic proximity of significantly different metabolites (VIP > 1, P < 0.05), visualized in chord and network diagrams derived from the correlation matrix (**Figures 4–M**). Various metabolites coordinate their functions within biological systems, and KEGG pathway analysis further elucidated their biological roles. Before KEGG pathway annotation, we merged differential metabolites identified from both ion modes (**Data S10**). To facilitate observation of each differential metabolite expression within the KEGG metabolic pathways, we selected pathways featuring more than five differential metabolites and represented them in a heatmap (**Supplementary Figure S6**, **Data S11**). KEGG enrichment analysis indicated significant enrichment in the amino acid biosynthesis pathway (**Figure 4**). Finally, a Differential Abundance Score pathway-based analysis captured overall metabolic changes, as presented in **Figure 4**.

## 4 Discussion

With the rising prevalence of obesity and an aging population, the incidence of diabetes has surged, posing a significant public health challenge (21). Individuals with diabetes frequently encounter a myriad of complex complications, including chronic pain stemming from diabetic ulcers and foot conditions, substantially elevating the risks of limb amputation and mortality (22). Unlike typical wounds, diabetic ulcers often progress to chronic, non-healing lesions owing to a multifaceted inflammatory microenvironment (23), characterized by factors like excessive ROS accumulation (24) and hypoxia (25). These elements collectively disrupt skin regeneration. Moreover, the impaired vasculature associated with diabetic wounds restricts blood flow, reducing oxygen supply and exacerbating inflammation around the ulcer (26). Despite available treatments for diabetic ulcers, including debridement, growth factor therapy, and topical antimicrobials, effective wound healing remains challenging (27). Further investigation into the molecular mechanisms underlying these processes may uncover potential therapeutic targets for enhancing wound healing in patients with diabetes.

Integrating NHANES database, transcriptomics, proteomics, and metabolomics has provided profound insights into the complex mechanisms underlying diabetic ulcer pathogenesis. In this study, we used normal and diabetic ulcer mice models (n = 4 for transcriptomics, n = 5 for proteomics, and n = 7 for metabolomics) to conduct a comprehensive analysis across these multi-omics platforms, aiming to uncover new dimensions in the healing landscape of DFUs. It is noteworthy that several estimates presented in **Table 3** exhibit wide confidence intervals (e.g., TyG-WC Q4: 95% CI, 0.10–1.77), suggesting potential instability in the effect sizes. This variability may, in part, reflect heterogeneity in baseline metabolic profiles (e.g., BMI, glycemic status) and lifestyle factors (e.g., dietary patterns, physical activity) among the study population. Despite the implementation of stratified analyses and multivariable adjustments to account for known covariates, the influence of unmeasured confounders—such as genetic predispositions or varying environmental exposures—cannot be fully excluded. Furthermore, while standard metabolic variables (e.g., age, sex, smoking) were incorporated into the model, certain relevant factors—including indices of insulin resistance and systemic inflammation—were not comprehensively adjusted due to missing data. These limitations may have contributed to residual confounding, thereby broadening the confidence intervals. Future investigations should prioritize subgroup analyses to mitigate population-level heterogeneity and incorporate a more robust panel of metabolic and inflammatory biomarkers to enhance model stability and interpretability.

Pathway analysis of the transcriptomic data revealed that significantly altered signaling pathways were predominantly associated with endothelial cell-mediated inflammatory responses, particularly the NF-κB signaling pathway, cytokine–cytokine receptor interactions, and TNF signaling pathway. Various herbal extracts can enhance wound healing by modulating the NF-κB pathway. For instance, balsam pears facilitate diabetic ulcer healing in mouse models by influencing the RAGE/NF-κB and VEGF/VCAM-1/eNOS signaling pathways (28). Additionally, scutellarein inhibits NF-κB-mediated luciferase expression and nuclear translocation, while reducing the phosphorylation of upstream signaling enzymes. The significant suppression of Src kinase activity further underscores its potential as an anti-inflammatory agent (29). The TNF pathway is crucial in initiating and progressing inflammatory and autoimmune disorders, cancer, and cardiovascular conditions (27, 30).

Regarding cytokine–cytokine receptor interactions, miRNA-497 treatment, both *in vivo* and *in vitro*, can reduce levels of pro-inflammatory cytokines such as IL-1β, IL-6, and TNF-α, thereby promoting DFU healing (31). In our investigation, we identified the pro-inflammatory gene *IGFN1* as significantly differentially expressed in both the diabetic and normal ulcer mouse models. *IGFN1* was initially characterized as a protein fragment using a yeast two-hybrid assay, with the KY protein as bait (32). *IGFN1-*deficient clones exhibit a reduced fusion index and pronounced morphological differences compared to C2C12 myotubes, indicating *IGFN1*’s role in myoblast fusion and differentiation (33). Immunohistochemistry confirmed elevated *IGFN1* expression in the diabetic ulcer model compared to the normal, suggesting that *IGFN1* may be a critical target in diabetic ulcers. Consequently, further exploring the pathways through which *IGFN1* influences these changes could have significant implications for diabetic ulcer prognosis.

Proteomics offers a powerful approach for elucidating the molecular mechanisms involved in disease progression, utilizing high-throughput, precise, sensitive, and reproducible techniques. TMT coupled with LC-MS/MS enables effective protein identification and quantification in tissue samples (34). In our proteomic analysis, the results showed significant enrichment of proteins related to DCM, oxidative phosphorylation, the PPAR signaling pathway, and the HIF-1 signaling pathway, suggesting the potential roles of these proteins in diabetic ulcers. Advanced DCM is often accompanied by severe PAD. However, effective therapeutic targets or treatments remain lacking for patients with these conditions. Therefore, discovering effective targets for DFUs may also improve DCM prognosis and treatment, representing important clinical significance. Diabetes-induced oxidative stress continually damages endothelial cells and impairs wound healing (35). The significance of HIF-1 inhibition, first noted in diabetic wound healing (36), is associated with compromised wound healing in diabetes. Enhancing HIF-1 activity has been shown to facilitate wound healing by promoting angiogenesis, proliferation, and migration of fibroblasts in diabetic mouse models (37, 38). Deferoxamine, an iron-chelating agent commonly used for treating iron overload, can mitigate oxidative stress and activate HIF-1, thus expediting diabetic wound healing (37). A recently optimized topical drug delivery system is set to undergo clinical trials (ClinicalTrials.gov registration no. NCT03137966) to evaluate its effectiveness in patients with DFUs. Notably, while HIF-1 signaling plays an important role in diabetic ulcers, a direct link between the PPAR signaling pathway and diabetic ulcers remains unexplored, presenting an avenue for future research.

Recent research has increasingly recognized that diabetes mellitus (DM) and its complications involve profound metabolic disturbances, which may be driven directly by hyperglycemia or occur independently through complex regulatory networks (39, 40). As an integrative reflection of both endogenous and exogenous factors—including genomic, transcriptomic, and proteomic layers—metabolomics offers unique insights into disease-specific molecular phenotypes. In particular, metabolic reprogramming has emerged as a critical interface between energy homeostasis and immune-inflammatory responses (41, 42). In our metabolomic analysis of DFUs, we observed significant enrichment in pathways related to amino acid biosynthesis, notably involving arginine, glutamine, and tryptophan. These amino acids are increasingly recognized as immunomodulatory metabolites. Arginine, for instance, serves as a substrate for arginase-1 (Arg1), promoting M2 macrophage polarization and suppressing chronic inflammation via inhibition of NF-κB–mediated secretion of proinflammatory cytokines such as TNF-α and IL-6 (43). Glutamine, a key energy source for lymphocytes and neutrophils, modulates immune function through the mTOR signaling axis; its depletion has been associated with impaired immune responses and enhanced inflammatory cascades (44). Tryptophan catabolism yields kynurenine, a ligand for the aryl hydrocarbon receptor (AhR), which has been shown to suppress NLRP3 inflammasome activation and mitigate oxidative stress (45).

Consistent with these findings, our data revealed decreased glutamine levels and increased kynurenine accumulation in DFU samples, suggesting that altered amino acid metabolism contributes to immune dysregulation and chronic inflammation in diabetic wound microenvironments. Moreover, metabolic stress induced by hyperglycemia and insulin resistance leads to mitochondrial dysfunction and shifts in cellular energy demands. Under such stress, hypoxia-inducible factor-1α (HIF-1α) is activated, promoting the transcription of glycolytic enzymes (e.g., LDHA) while simultaneously repressing oxidative phosphorylation (46). Notably, our pathway analysis revealed concurrent enrichment of HIF-1 signaling and amino acid biosynthesis pathways, implying a coordinated metabolic adaptation that enables immune cells to function in hypoxic, nutrient-deprived settings characteristic of chronic wounds. Taken together, these results highlight the central role of amino acid metabolic reprogramming in orchestrating inflammatory and energy responses during DFU pathogenesis.

Recent studies have further revealed the dynamic characteristics of metabolic reprogramming in DFU. Related studies have shown that metabolomics has found significant accumulation of phenylpyruvate in DFUs. Increased phenylpyruvate impairs wound healing and enhances inflammatory responses, while reducing phenylpyruvate by restricting phenylalanine in the diet can alleviate uncontrolled inflammation and benefit diabetic wounds (47). There are also studies showing that TMAO accumulates in diabetic wounds, causing macrophages to persist in the pro-inflammatory M1 phenotype, hindering tissue repair. TMAO-targeted anti-inflammatory agents can reprogram macrophage metabolism by inhibiting the IRE1α/XBP1/HIF-1α signaling pathway, prompting it to switch to anti-inflammatory M2, while improving angiogenesis and reducing oxidative stress (48). These advances together support the core conclusion of this study—the metabolism-inflammatory axis is a key target for the treatment of DFU.

Meanwhile, results from the NHANES cohort revealed a significant positive association between TyG-WC/BMI and the risk of non-healing lower extremity ulcers (NHLU). TyG-WC and TyG-BMI have been recognized as sensitive indicators of insulin resistance and visceral obesity, with elevated levels reflecting lipid metabolic disorders and a state of chronic low-grade inflammation (49, 50). Accumulation of visceral adipose tissue has been reported to promote the secretion of pro-inflammatory cytokines such as IL-6, thereby activating the NF-κB signaling pathway, impairing fibroblast migration and angiogenesis, and ultimately delaying ulcer healing (51–53). n our diabetic mouse model, we observed significant upregulation of NF-κB–related genes, including TNF and IL1B, consistent with the inflammatory phenotype observed in patients with elevated TyG-WC/BMI. These findings suggest that TyG-WC/BMI may serve as a potential biomarker for identifying individuals at high risk for NF-κB pathway overactivation and may aid in guiding the use of anti-inflammatory therapeutic strategies, such as JAK/STAT inhibitors.

This study also has a few limitations, the study relies on diabetic ulcer mouse models, which, while valuable, may not fully replicate the complexity of diabetic ulcers in humans, limiting the direct applicability of findings.

## 5 Conclusion

This study emphasizes the complexity and serious implications of diabetic ulcers, highlighting their association with significant comorbidities and elevated mortality rates. The integration of high-throughput multi-omics techniques—transcriptomics, proteomics, and metabolomics—enabled the development of a compound-reaction-enzyme-gene network that elucidates the underlying molecular mechanisms of diabetic ulcers. This comprehensive molecular map provides valuable insights into diabetic ulcer pathogenesis and facilitates the identification of novel therapeutic targets and strategies for improving patient outcomes.

## Data Availability

The contributions originally presented in this study are included in the article and supplementary material. Further inquiries can be directed to the corresponding authors.
