# Supplementary material for: Integrated transcriptomic, proteomic, and metabolomic analysis unveils key roles of protein and nucleic acid interactions in diabetic ulcer pathogenesis
Source: Front Endocrinol (Lausanne). 2025 Jun 20;16:1574858. doi: 10.3389/fendo.2025.1574858 (PMC12226305; doi:10.3389/fendo.2025.1574858)
Supplement: Supplementary file 1 [file DataSheet1.zip › Supplementary file 9.DOCX]

***Supporting information***

**Integrated Transcriptomic, Proteomic, and Metabolomic Analysis Unveils Key Roles of Protein and Nucleic Acid Interactions in Diabetic Ulcer Pathogenesis**

**Yongpan Lu1,2†, Hairui Gao1†, Sen Wang3†, Han Xu4, Zhiyu Chen4, Yixin Zhang5*, Yunfei Gu6*, Xiaomei Sun1***

^1^Department of Anorectal Surgery, The Affiliated Hospital of Qingdao University, Qingdao, Shandong, 266000, China.

^2^Department of Plastic Surgery, The First Affiliated Hospital of Shandong First Medical University & Shandong Provincial Qianfoshan Hospital, Jinan, Shandong, 250014, P. R. China.

^3^Department of Ultrasound, Beijing Tiantan Hospital, Capital Medical University, Beijing, 100050, China.

^4^Department of Radiology，Qilu Hospital of Shandong University, Jinan 250012, China.

^5^Department of Ultrasound, Sun Yat-sen Memorial Hospital, Sun Yat-sen University, Guangzhou, Guangdong, 510120, China.

^6^Department of Colorectal surgery, The Affiliated Hospital of Nanjing University of Chinese Medicine, Jiangsu Province Hospital of Chinese Medicine, the First Clinical Medical College, Nanjing, Jiangsu, 210029, China.

†These authors contributed equally to this work.

*These authors have contributed equally to this work.

* Corresponding authors:

Xiaomei Sun, M.D.

Department of Anorectal, The Affiliated Hospital of Qingdao University, Qingdao, Shandong, 266000, China.

Email addresses for correspondence:

sunxiaomei@qdu.edu.cn

***Supplementary Figures***


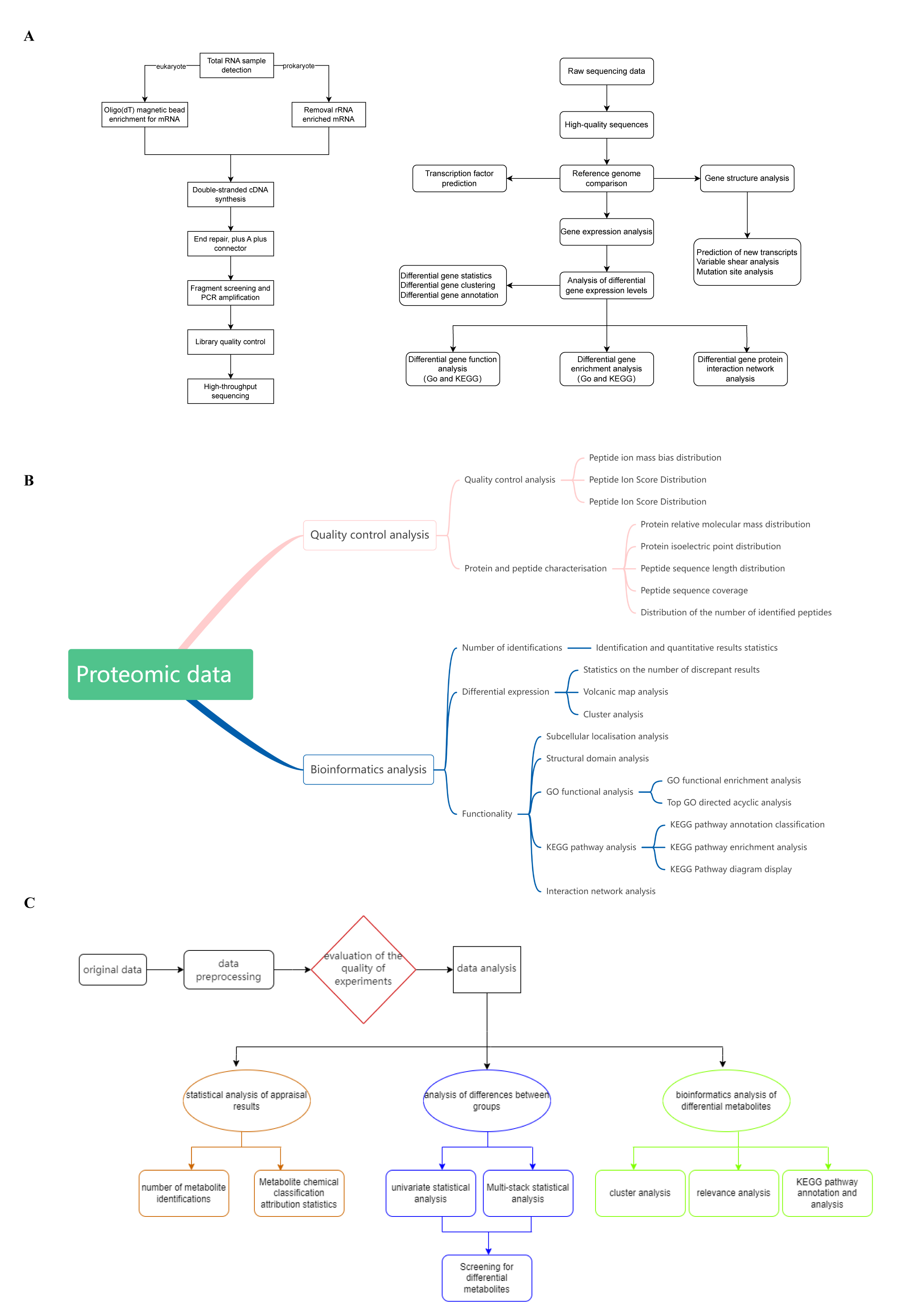


**Supplementary Figure 1.** **(A)** The flowchart delineating the transcriptomic data analysis conducted in our study. **(B)** The flowchart illustrating the proteomic data analysis employed in our research. **(C)** The flowchart representing the metabolomic data analysis carried out in our investigation.

**Supplementary Figure 2.** The frequency and length of each mutation based on the identified SNP sites.

**Supplementary Figure 3.** Statistics of the spectra, peptides, and proteins obtained in this study.

**
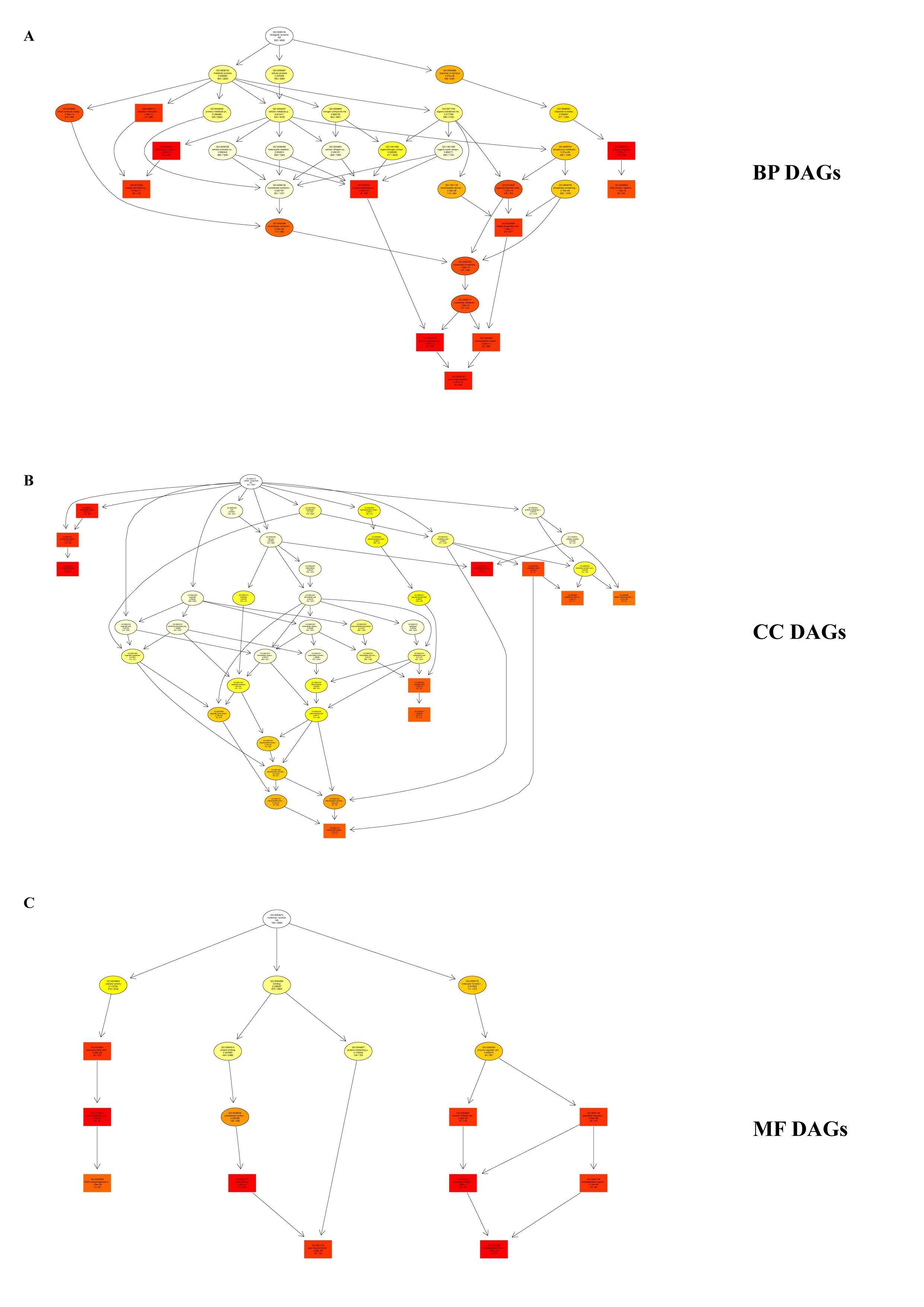
**

**Supplementary Figure 4.** For each of the three major GO categories, the top 10 most enriched ones were selected as the main nodes of the directed acyclic graph, represented by boxes. **(A)** PST_vs_PC group GO enrichment directed acyclic graph (Biological Process, BP). **(B)** PST_vs_PC group GO enrichment directed acyclic graph (Cellular Component, CC). **(C)** PST_vs_PC group GO enrichment directed acyclic graph (Molecular Function, MF).

**Supplementary Figure 5.** Examples of KEGG pathway diagram. **(A)** The PPAR signaling KEGG pathway diagram. **(B)** The HIF-1 signaling KEGG pathway diagram. The red boxes in the figure indicate that the DEPs are all upregulated, and the green boxes indicate that the DEPs are all downregulated. Small circles represent small molecule metabolites, and large circles represent other pathways.

**Supplementary Figure 6.** KEGG pathway differential metabolite clustering heatmap. Each row in the figure represents a differential metabolite (i.e., the vertical axis represents the metabolites with significant differential expression), and each column represents a group of samples (i.e., the horizontal axis represents sample information). Red represents significant upregulation, and blue represents significant downregulation. The depth of color indicates the degree of upregulation and downregulation. Metabolites with similar expression patterns are clustered in the same cluster on the left.

**Note：All figures and their corresponding supplementary datasets as follows:**

**Figure S3 –** Supplementary Data Sheets 1 and 2

**Figure S6 –** Supplementary Data Sheet 11

***Supplementary Data***

**Data S1. Protein Identification List.**

This table lists all identified proteins based on LC-MS/MS analysis. Protein identification was performed using the UniProt database, with a 1% false discovery rate (FDR) threshold at both the peptide and protein levels. Only proteins with at least two unique peptides are included.

**Data S2. Peptide identification list.**

Contains all identified peptides corresponding to proteins listed in Data S1. Information includes peptide sequence, charge state, retention time, and confidence score. A 95% confidence threshold was used for peptide filtering.

**Data S3. Analysis of the significant differences between PST VS PC.**

Summarizes proteins with statistically significant abundance differences between PST and PC groups. Statistical significance was determined using two-tailed Student’s t-tests followed by Benjamini-Hochberg correction for multiple comparisons. Results include adjusted p-values and log2 fold changes.

**Data S4. Subcellular localization analysis.**

Provides predicted subcellular localization of significantly altered proteins using WoLF PSORT and CELLO tools. Locations include nucleus, cytoplasm, mitochondria, and plasma membrane, among others.

**Data S5. Gene Ontology Resource.**

Includes GO annotation results for all identified proteins. GO terms are categorized into biological processes, cellular components, and molecular functions. Annotation was performed using the Gene Ontology Resource.

**Data S6. KEGG pathway analysis.**

Lists enriched KEGG pathways derived from the differentially expressed protein dataset. Pathway significance was assessed using Fisher’s exact test with Benjamini-Hochberg FDR correction.

**Data S7. Metabolite Qualitative and Quantitative Results.**

Presents qualitative and quantitative data of metabolites detected using LC-MS/MS under both positive and negative ion modes. Includes retention time, m/z values, and concentration levels.

**Data S8. 8a. PCA model parameters in positive ion mode**

**8b. PCA model parameters in negative ion mode**

**8c. PLS-DA model parameters in positive ion mode**

**8d. PLS-DA model parameters in negative ion mode**

**8e. OPLS-DA model parameters in positive ion mode**

**8f. OPLS-DA model parameters in negative ion mode**

This dataset provides model performance metrics including R2X, R2Y, and Q2 values, used to evaluate the quality and predictive power of the multivariate models.

**Data S9. Sample NEGPOS Qualitative.**

Shows qualitative distribution and relative intensity of metabolites across sample groups under both ion modes. Useful for group-based comparison of metabolic profiles.

**Data S10. Metabolism KEGG.**

Displays mapping of identified metabolites to KEGG metabolic pathways. Provides metabolite IDs, compound names, and matched pathway identifiers.

**Data S11. Metabolic enrichment.**

Lists significantly enriched metabolite pathways based on statistical enrichment analysis. Includes pathway name, matched metabolites, enrichment score, and adjusted *p*-values.

**Data S12. Heatmap Data for Sample Clustering (TST vs. TC)**

Provides normalized expression data used to generate the hierarchical clustering heatmap of selected metabolic or proteomic features across experimental groups.

**Data S13. Gene Ontology Enrichment Analysis.**

Details significant GO terms enriched among differentially expressed genes or proteins. Includes GO ID, term name, enrichment score, gene count, and FDR-adjusted *p*-values.

**Data S14. KEGG enrichment results.**

Lists significantly enriched KEGG pathways from transcriptomic or proteomic analyses. Provides pathway ID, name, gene counts, enrichment ratios, and adjusted significance values.

**Note：All figures and their corresponding supplementary datasets as follows:**

**Figure 3A –** Supplementary Data Sheet 3

**Figure 3D –** Supplementary Data Sheet 4

**Figure 3H –** Supplementary Data Sheet 5

**Figure 3J –** Supplementary Data Sheet 6

**Table 5 –** Supplementary Data Sheet 7

**Figure 4G –** Supplementary Data Sheets 8

**Figure 4I –** Supplementary Data Sheet 9

**Figure 4K–M –** Supplementary Data Sheet 10

**Figure 2F –** Supplementary Data Sheet 12

**Figures 2H and 2I –** Supplementary Data Sheet 13

**Figures 2K and 2L –** Supplementary Data Sheet 14
