# Supplementary figures and images for: Integrated transcriptomic, proteomic, and metabolomic analysis unveils key roles of protein and nucleic acid interactions in diabetic ulcer pathogenesis
Source: Front Endocrinol (Lausanne). 2025 Jun 20;16:1574858. doi: 10.3389/fendo.2025.1574858 (PMC12226305; doi:10.3389/fendo.2025.1574858)

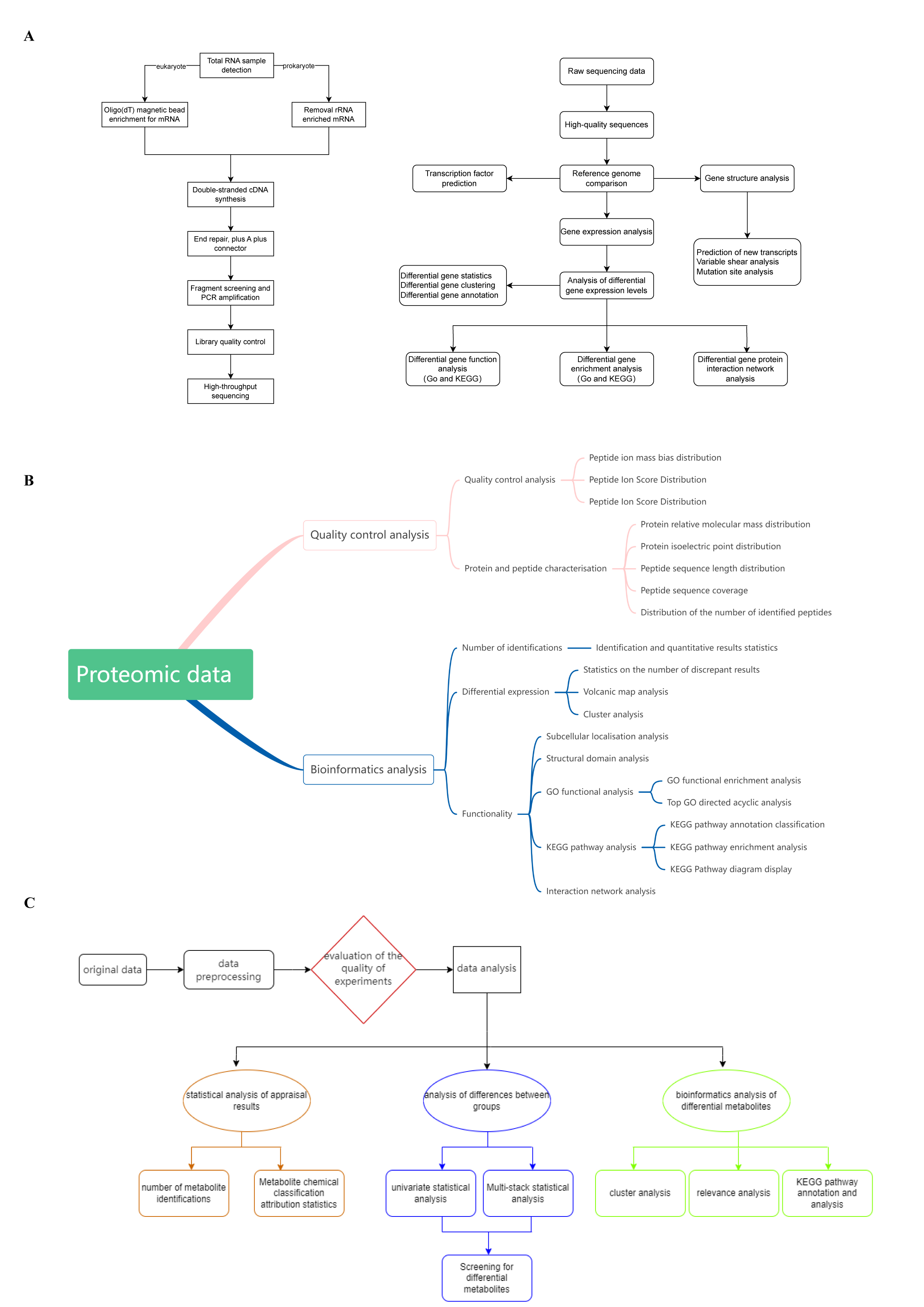

Supplement: Supplementary file 1 [file DataSheet1.zip › Supplementary file 3.JPEG]

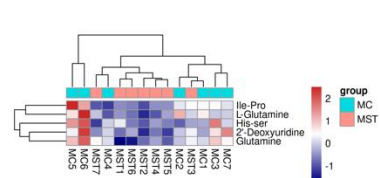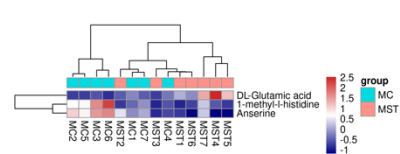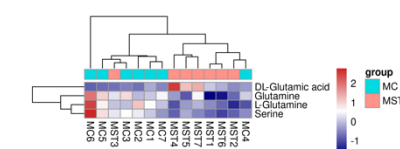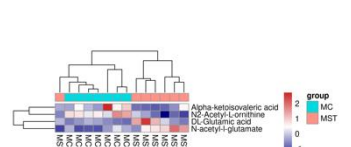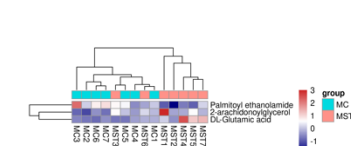

Supplement: Supplementary file 1 [file DataSheet1.zip › Supplementary file 8.PDF]

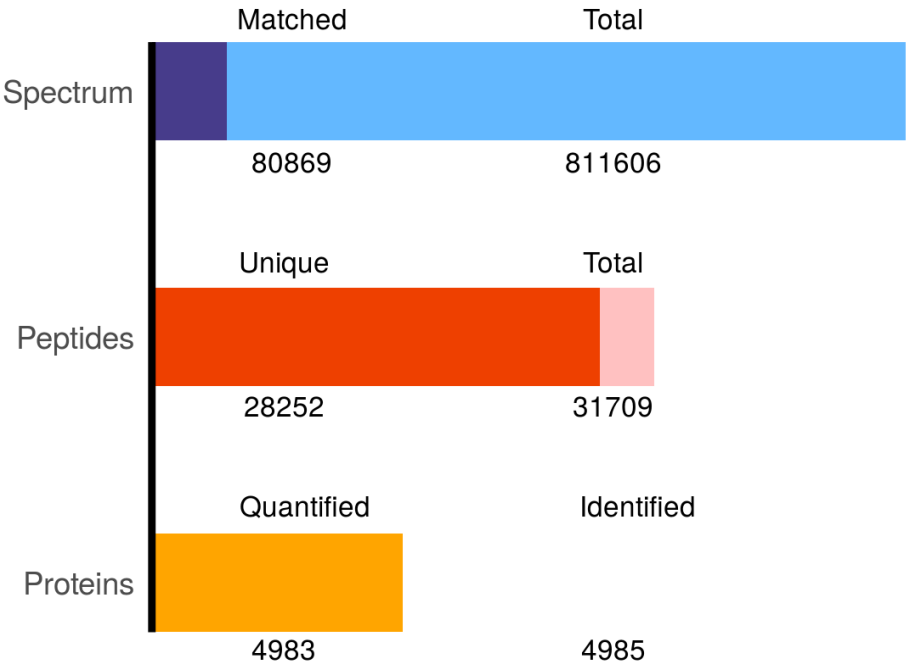

Supplement: Supplementary file 1 [file DataSheet1.zip › Supplementary file 5.PDF]

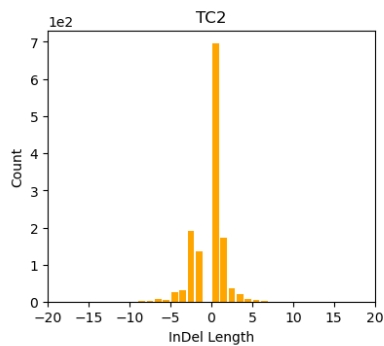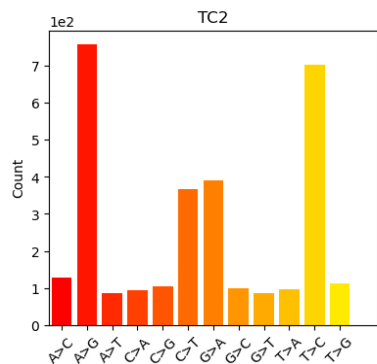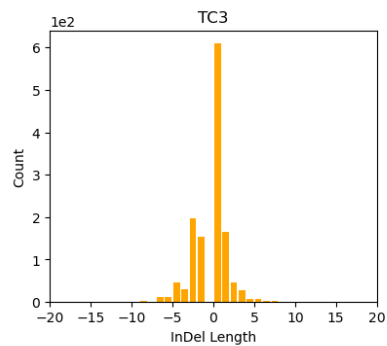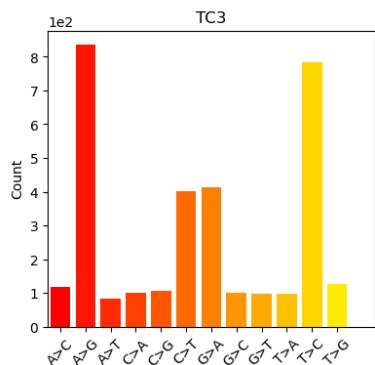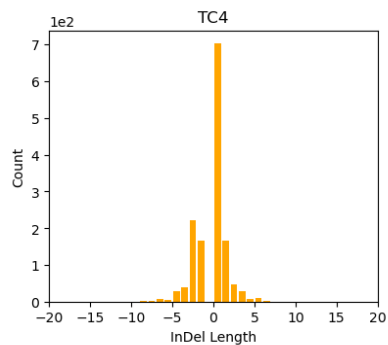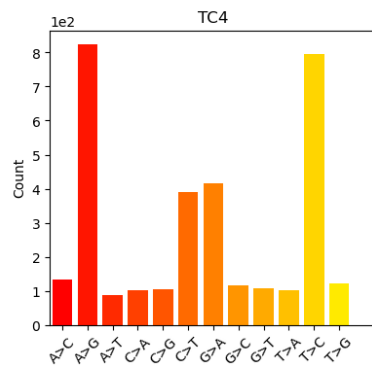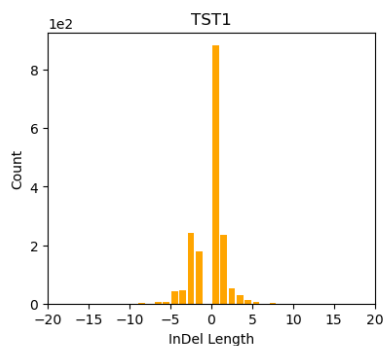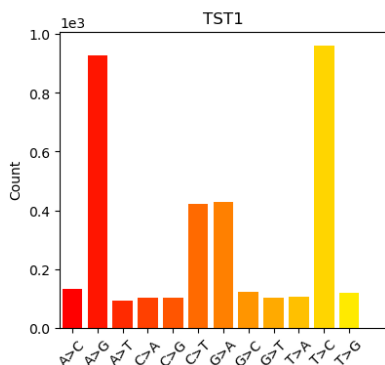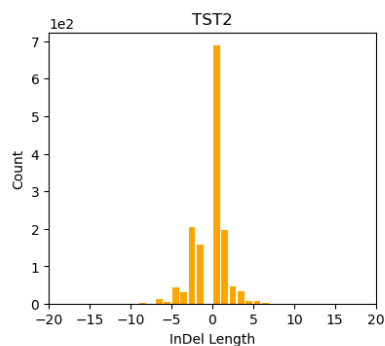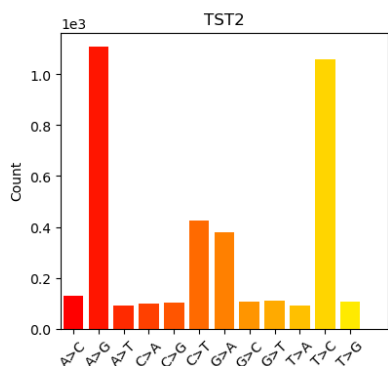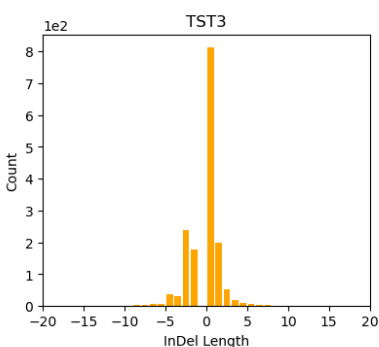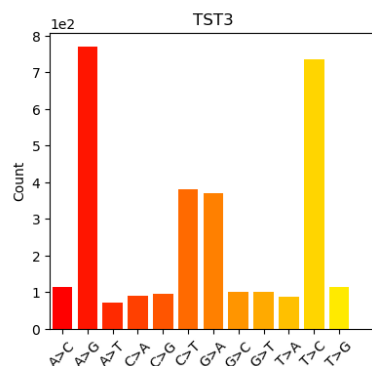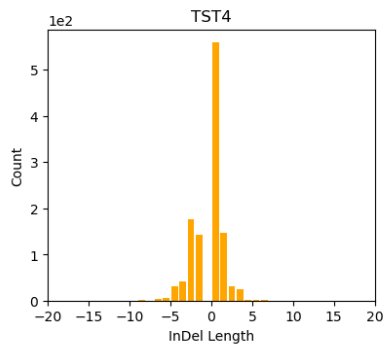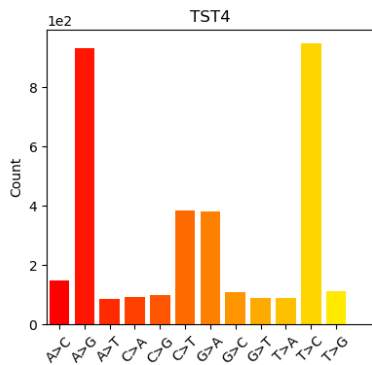

Supplement: Supplementary file 1 [file DataSheet1.zip › Supplementary file 4.PDF]

A

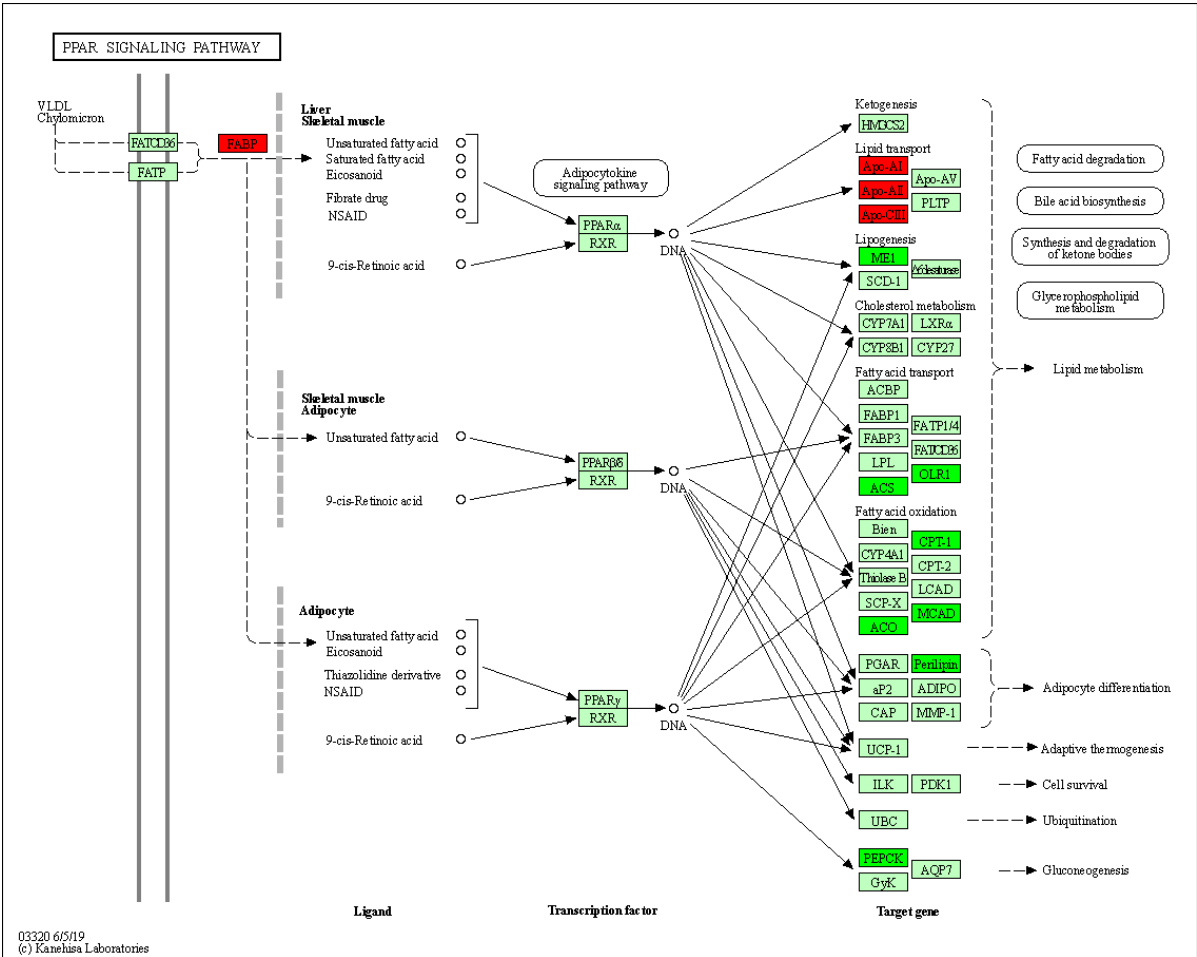

B

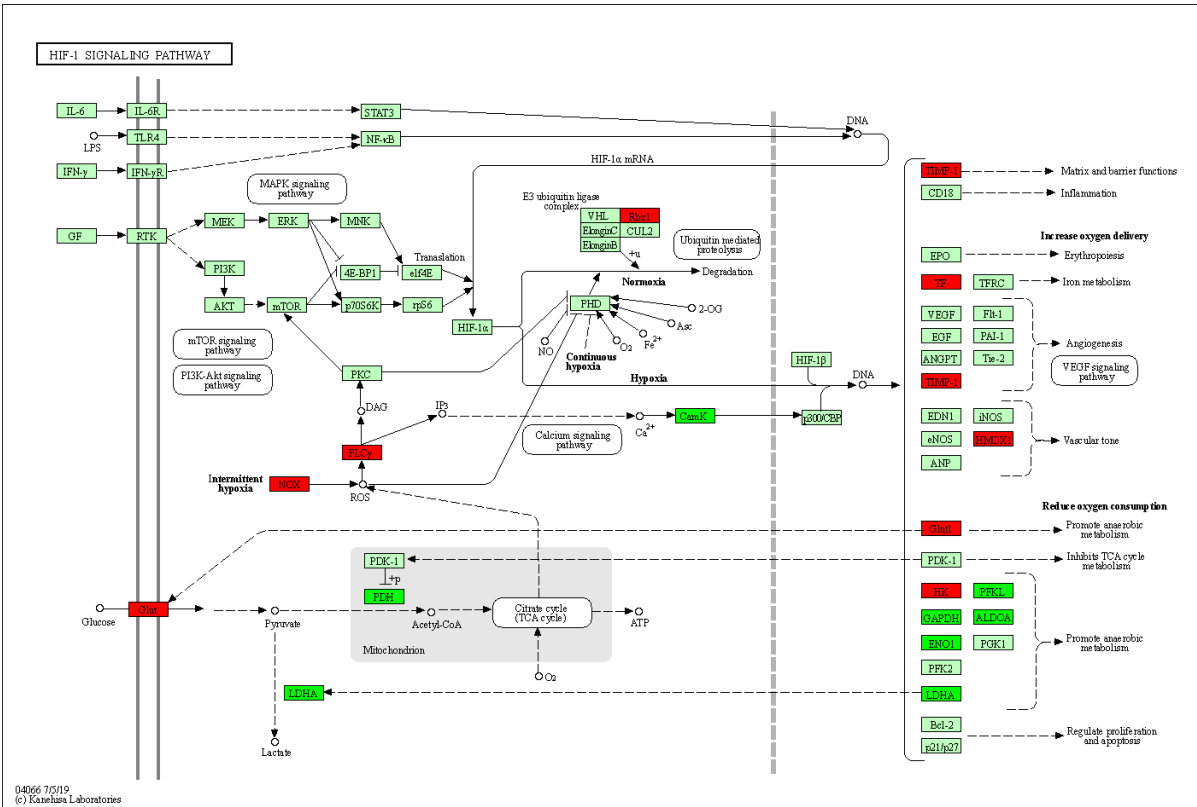

Supplement: Supplementary file 1 [file DataSheet1.zip › Supplementary file 7.PDF]

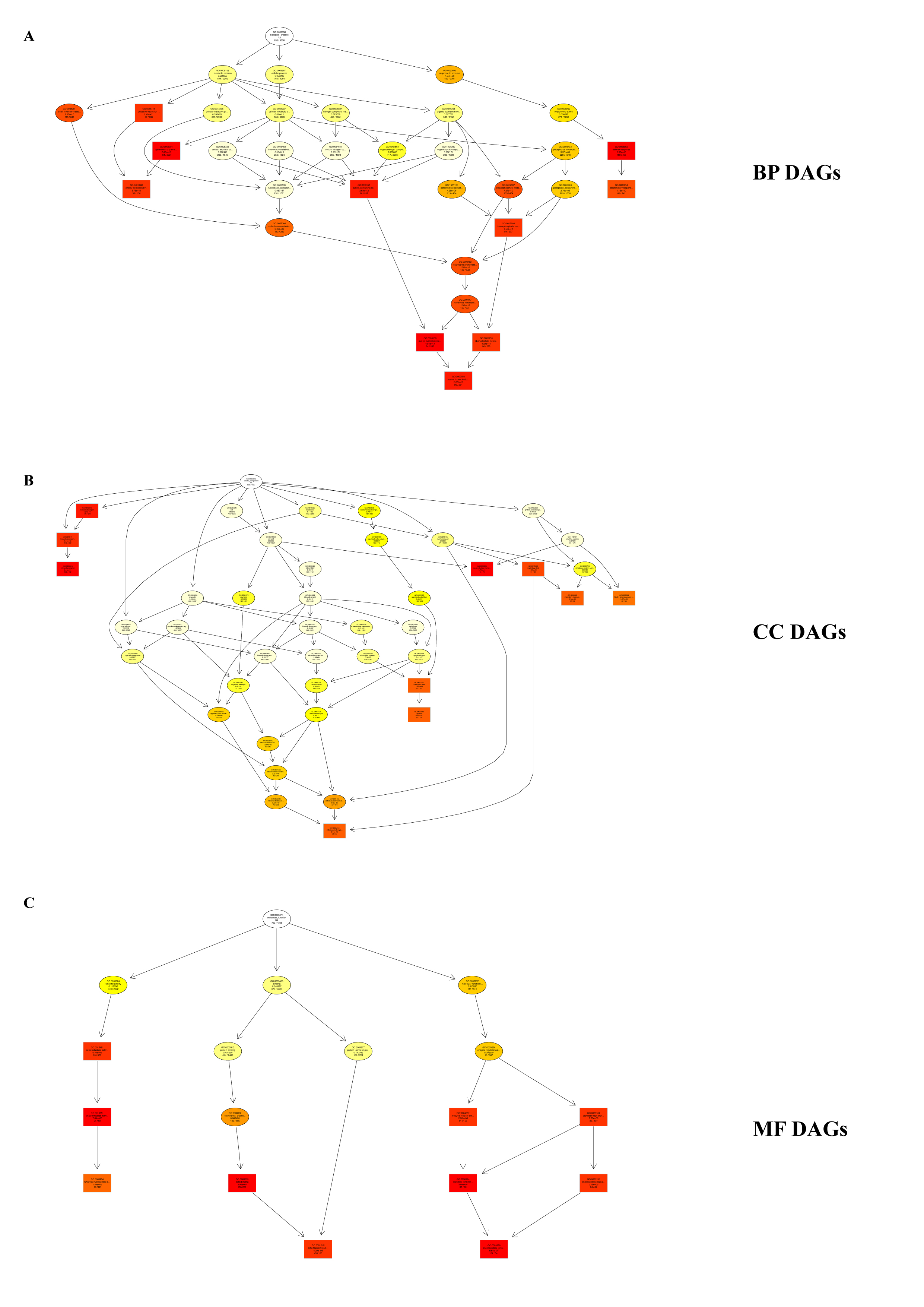

Supplement: Supplementary file 1 [file DataSheet1.zip › Supplementary file 6.JPEG]
